# All-in-one theranostic nano-platform based on polymer nanoparticles for BRET/FRET-initiated bioluminescence imaging and synergistically anti-inflammatory therapy for ulcerative colitis

**DOI:** 10.1186/s12951-022-01299-8

**Published:** 2022-03-02

**Authors:** Xiangji Yan, Chunhua Yang, Mei Yang, Yana Ma, Yuanyuan Zhang, Yujie Zhang, Cui Liu, Qiuran Xu, Kangsheng Tu, Mingzhen Zhang

**Affiliations:** 1 School of Basic Medical Sciences, Xi’an Key Laboratory of Immune Related Diseases, Xi’an Jiaotong University, Xi’an, 710061 Shaanxi China; 2 Key Laboratory of Environment and Genes Related to Diseases, Xi’an Jiaotong University, Ministry of Education, Xi’an, 710061 Shaanxi China; 3 Institute for Biomedical Sciences, Center for Diagnostics and Therapeutics, Digestive Disease Research Group, Georgia State University, Atlanta, 30302 GA USA; 4 Laboratory of Tumor Molecular Diagnosis and Individualized Medicine of Zhejiang Province, Zhejiang Provincial People’s Hospital, Affiliated People’s Hospital, Hangzhou Medical College, Hangzhou, 310014 Zhejiang China; 5 Department of Hepatobiliary Surgery, The First Affiliated Hospital of Xi’an Jiaotong University, Xi’an, 710061 Shaanxi China

**Keywords:** Ulcerative colitis, PLGA, Inflammation imaging, P-selectin, Theranostic

## Abstract

**Background:**

Ulcerative colitis (UC), a subtype of inflammatory bowel disease (IBD), has evolved into a global burden given its high incidence. There is a clinical need to create better diagnostic and therapeutic approaches to UC.

**Results:**

We fabricated P-selectin binding peptide-decorated poly lactic-co-glycolic acid (PBP-PLGA-NP) doped with two lipophilic dyes, DiL and DiD. Meanwhile, two low-toxic anti-inflammatory natural products (betulinic acid [BA] and resveratrol [Res]) were co-loaded in the PBP-PLGA-NP system. The BA/Res-loaded NPs had an average size of around 164.18 nm with a negative zeta potential (− 25.46 mV). Entrapment efficiencies of BA and Res were 74.54% and 52.33%, respectively, and presented a sustained drug release profile. Further, the resulting PBP-PLGA-NP could be internalized by RAW 264.7 cells and Colon-26 cells efficiently in vitro and preferentially localized to the inflamed colon. When intravenously injected with luminol, MPO-dependent bioluminescence imaging to visualize tissue inflammation was activated by the bioluminescence and fluorescence resonance energy transfer (BRET-FRET) effect. Importantly, injected NPs could remarkably alleviate UC symptoms yet maintain intestinal microbiota homeostasis without inducing organ injuries in the mice models of colitis.

**Conclusions:**

This theranostic nano-platform not only serves as a therapeutic system for UC but also as a non-invasive and highly-sensitive approach for accurately visualizing inflammation.

**Graphical Abstract:**

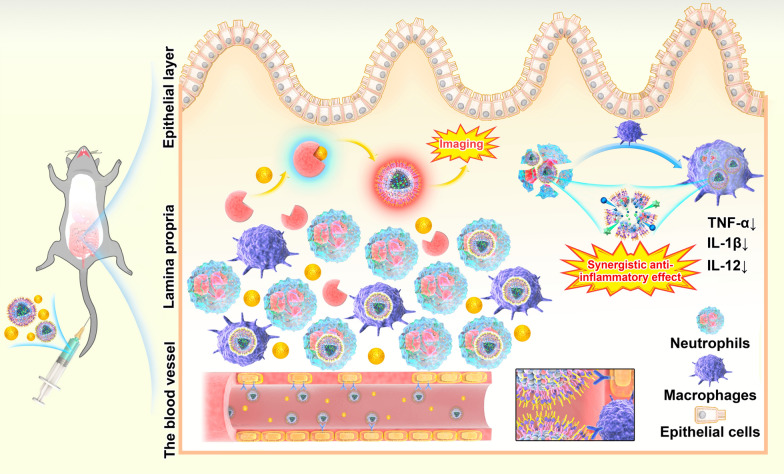

**Supplementary Information:**

The online version contains supplementary material available at 10.1186/s12951-022-01299-8.

## Background

Ulcerative colitis (UC) is a chronic, nonspecific inflammatory disease of unclear aetiology, and its incidence has been rapidly increasing in recent years [1–4]. The etiology of UC is not fully understood, which is the result of the comprehensive action of genetic, immune factors and environmental [5–7]. Some traditional anti-inflammatory drugs, such as 5-aminosalicylic acid (5-ASA), thiopurines, and corticosteroids, can treat mild-to-moderate UC, but the common problem of anti-inflammatory drugs is inevitable side effects caused by systemic exposure [8, 9]. To overcome these obstacles, researchers had developed a large number of nanoparticles (NPs)-based drug delivery systems (NDDSs) for UC treatment, such as liposomes, micelles, and metal-organic frame [10–12]. NDDSs are expected to deliver drugs exclusively and precisely to the inflammatory areas. One limitation of most current NDDSs is that they are only focusing on UC therapy; thus, the use of these NDDSs relies heavily on the time-consuming UC diagnosis [13]. There is a great need to develop novel theranostic carrier systems for concomitant UC diagnosis and therapy.

Nowadays, the diagnosis of UC mainly depends on clinical manifestations and auxiliary examinations, among which endoscopy is the most used method [14, 15]. Due to endoscopy’s high cost and inconvenience, other substitutions, such as biomarker-associated imaging, are developed quickly. In the inflammatory progress of UC, the heme-containing enzyme myeloperoxidase (MPO) gradually increases in neutrophils, macrophages, and monocytes [16–18]. MPO catalyzes the generation of a series of cytotoxic reactive species, such as aldehydes, hypochlorous acid, and hydroxyl radicals [19], which can induce tissue oxidative damage and promote inflammation [20, 21]. Therefore, MPO can be used as an indicator of inflammation to diagnose and monitor the process of inflammation by obtaining the production of MPO [22, 23]. The current methods for detecting MPO are not suitable for the detection of deep tissues in vivo. Optical detection of MPO can be probed by luminol, a reagent that produces blue luminescence in vivo via an MPO-dependent process [24], which is more specific, safer, and reliable than other imaging methods. However, luminol-based MPO detection method can only detect superficial inflammation because of its short wavelength (λ_max_ = 425 nm), which makes a low tissue penetration rate, limiting its application in the deep tissue [25, 70]. Therefore, developing a method to shift the blue light emitted by luminol to the near infrared ray (NIR) range can expand the detection capability of luminol-based bioluminescence imaging for detecting MPO activity in deeper inflamed tissues.

Natural compounds offer an excellent source of anti-inflammatory drugs. Resveratrol (Res), a natural stilbenoid (one type of polyphenol), is found to be capable of ameliorating the severity of several diseases in animal models, including inflammatory bowel disease (IBD), cardiovascular disease, cancer, and ischemic injury [26–28]. Some studies have shown that Res can inhibit IBD and colitis-associated colon cancer in mouse models [29–31]. The clinical trial also demonstrated that Res decreased plasma TNF-*α* and NF-*κ*B, increased peroxide dismutase and total antioxidant capacity, and ameliorated intestinal inflammation in UC patients [32–34]. Through a literature search, we have noticed that the dietary triterpenoid betulinic acid (BA) could reduce the level of MPO and lipid hydroperoxide in the colon [35, 36]. BA also restored the catalase and superoxide dismutase in colitis mice, reduced the level of glutathione, and significantly attenuated the expression of inflammatory mediators such as matrix metalloproteinase-9 and prostaglandin E2 [37]. In addition, BA could also attenuate visceral pain induced by acetic acid and mustard oil in mice [38]. These outcomes seem complementary to the current effects of Res for UC, suggesting a combination of these two natural produces may offer a synergetic efficacy against UC.

Despite the promising effects, the major drawbacks of Res and BA are their low water solubility and poor oral absorption; both compromise their effectiveness. At present, a variety of nanocarriers have been developed for the loading and transport of anti-inflammatory drugs in the treatment of colitis, such as liposomes, polymer-carriers, inorganic nanoparticles, and protein nanoparticles [39–41]. Among them, polymer-carriers based on PLGA and PEGylated polymer (PLGA-PEG) have attracted wide attention. PLGA, as a drug delivery carrier, has been approved by FDA for clinical studies due to its biodegradability, excellent biocompatibility, and good sustained drug release [42, 43]. PLGA-PEG polymers not only make the NPs have good stability but also help the NPs avoid the reticuloendothelial system (RES) clearance, which makes the NPs have a long cycle time in vivo. In addition, the easy modification of PEG offered the extra targeting ability of NPs [44]. Importantly, PLGA-based carrier is particularly advantageous in delivering hydrophobic drugs. It can effectively improve the solubility of hydrophobic drugs and has high encapsulation efficiency to be used as the best delivery carrier of BA and Res.

P-selectin is a membrane glycoprotein expressed on vascular endothelial cells in most tissues, and is a member of the selectin family that mediates cell rolling and platelet adhesion. It plays an important role in the accumulation of white blood cells towards the damaged site in the early stage of inflammation [45–48]. Notably, elevated P-selectin expression was found in inflammatory sites of the ulcerative colon but not healthy tissues in humans and mice, suggesting that P-selectin may be a specific target for delivery of nanomedicine to the colon in the treatment of UC.

**Fig. 1:**
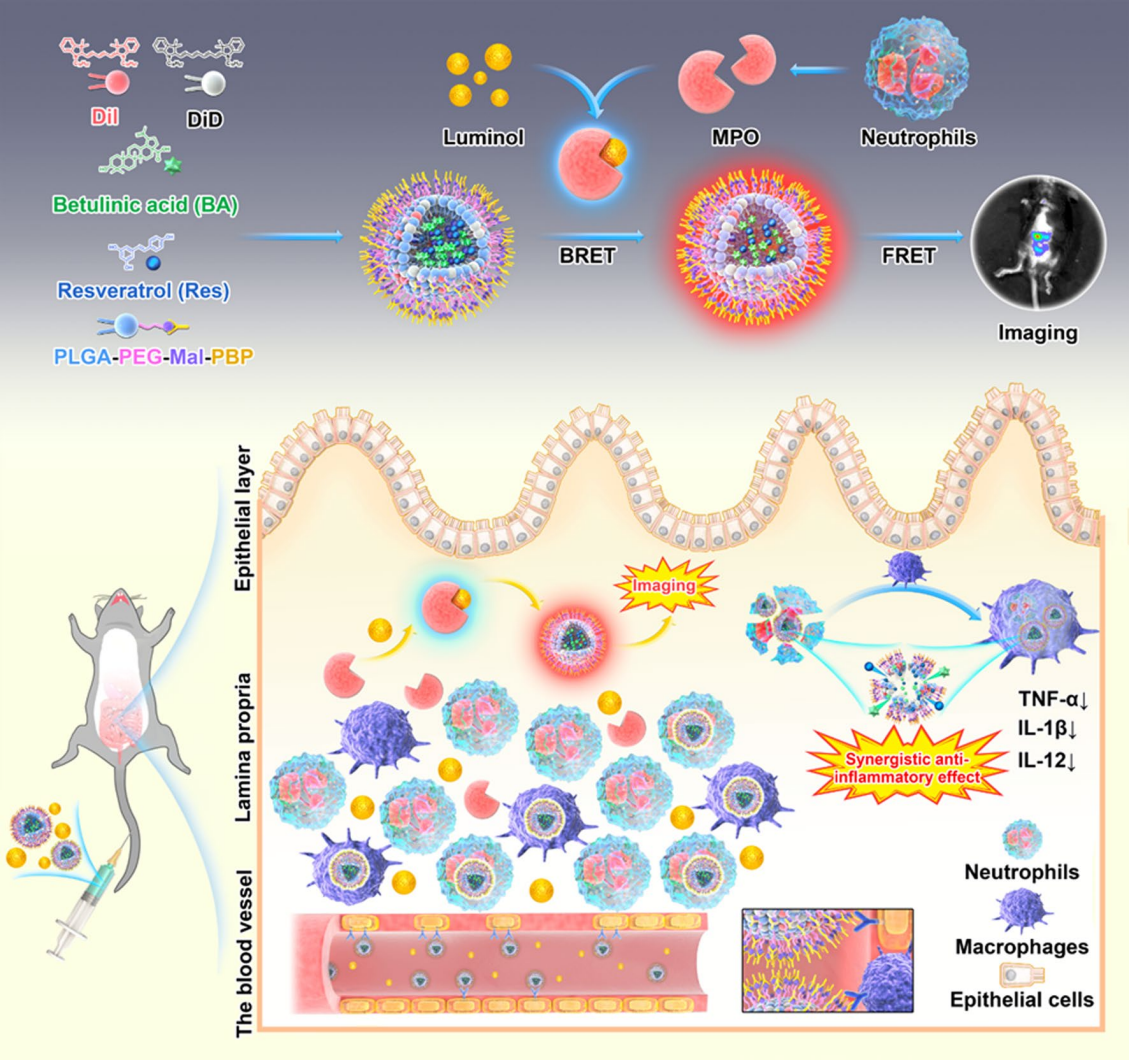
Preparation of BA/Res@NP-PBP and schematic diagram of targeted delivery of BA/Res@NP-PBP to the inflamed colon. BA and Res were encapsulated into the PLGA core and connected with PLGA-PEG-Mal and PBP. DiL and DiD were co-incorporated in the NPs. Neutrophils and macrophages in inflammatory sites highly expressed MPO, which could combine with luminol to generate blue light. This wavelength could be lengthened and red-shifted to 670 nm through BRET-FRET effect between DiL/DiD-loaded BA/Res@NP-PBP NPs and luminol. The schematic diagram showed an MPO-dependent inflammation imaging via the BRET-FRET effect and a synergistic therapeutic effect between BA and Res for UC

To design a BA and Res co-delivering, P-selectin-targeting NP to colon endothelial cells, we prepared surface P-selectin binding peptide (PBP) modified PLGA-NPs and incorporated them with two lipophilic dyes. We injected NPs together with luminol and expected that the BRET-FRET effect on NPs could enable MPO-dependent inflammation imaging in deep colitic tissues (Fig. 1). Furthermore, we evaluated the synergistic effect of the co-delivery of BA and Res (BA/Res@NP-PBP) versus separately delivered BA or Res by NPs in DSS-induced acute and chronic UC models.

## Materials and methods

### Materials

PLGA (wt: 38,000–54,000), poly (vinyl alcohol) (PVA, 86–89% hydrolyzed), PLGA-PEG-Mal (PEG MW 5000), and P-selectin binding peptide (PBP) were obtained from NSP-Functional polymers and copolymers (Winston-Salem, USA). Fluorescent dyes (DiL, DiD, and DiR) were obtained purchased from Promokine (Heidelberg, Germany). Colon-26 cells and Raw 264.7 cells were purchased from ATCC, USA and CLS Cell Lines Service, Germany.

### NPs preparation

Nanoparticles were synthesized by single microemulsion method. Dichloromethane (DCM) was used to dissolve the BA, Res, PLGA (50 mg), and PLGA-PEG-Mal in 2 mL of in specific proportions. An oil-in-water emulsion was formed by dropping the polymer solution dropwise into 2.5% polyvinyl alcohol (PVA) solution (4 mL). Then, the solution was submitted to a probe sonicator on an ice bath of 25% amplitude for 4 min (Branson S-450; Danbury, CT, USA). Then the solution was gradually added to water, and then the residual DCM is removed by rotary evaporation for 15 min. The solution was stirred for 4 h to make the NPs spherical. After stirring, centrifuged at 15,000 rpm for 15 min and poured off the supernatant. Resuspended the precipitate with deionized water and repeated centrifugation 3 times to purify the NP. Finally, the precipitate was suspended with a small amount of ionized water and added to the cryopreservation solution, which was stored at − 80 °C. Blank@NP and Dye-loaded NP were prepared in the same way.

### Characterization

Malvern Zetasizer Nano ZS90 Apparatus (Worcestershire, UK) was used to determine the size and zeta potential of BA/Res@NP and BA/Res@NP-PBP. Transmission Electron Microscopy (TEM) and Scanning Electron Microscope (SEM) were used to obtain the morphology of BA/Res@NP and BA/Res@NP-PBP.

The loading efficiency of BA and Res were evaluated using a high-performance liquid chromatography (HPLC) analysis via an Agilent® 1100 LC System (Agilent Technologies Inc.; CA, USA), which was performed at room temperature. The C18 column was used in the experiment. Dissolved the BA and Res loaded NPs with acetonitrile/water (90:10) or (70:30). Before the experiment, the liquid was filtered with a 0.45 μm filter. Between each sample, rinsed and balanced the C18 column. Set the detection wavelength of BA and Res at 207 and 324 nm, respectively. Made standard curves with BA and Res standard products.

The release ability of BA and Res from BA@NP, Res@NP, and BA/Res@NP was evaluated in reference to the experimental methods of others’ work [49]. Each dialysis bag (MW 10k) contained 5 mL of PBS (pH 6.5, 7.4, 8.0), dissolved 1 mg of the BA@NP, Res@NP, or BA/Res@NP. The external fluid of the dialysis bag was 100 mL of normal PBS. Stirred the liquid at 200 rpm. At specific time points (0, 1, 2, 4, 8, 12, 24, 32, and 48 h), removed 3 mL liquid out of the dialysis bag and added fresh PBS at the same pH to 100 mL. The concentration of BA and Res in the removed 3 mL liquid was measured with an ultraviolet spectrophotometer to determine the cumulative drug release.

### Cell culture

Cells were cultured in 5 cm^2^ culture dish and placed in an cell incubator with 37 °C, 5% CO_2_. Culture the Raw 264.7 cells with Dulbecco’s Modified Eagle Medium (DMEM), while the Colon-26 cells were with RPMI 1640 medium. Add 100 U/mL of penicillin and streptomycin and 10% heat-inactivated fetal bovine serum to the DMEM and RPMI 1640 medium. The cells were subcultured when the density of the culture dish was about 85%, and the Colon-26 cells were digested with trypsin.

### Cellular internalization of NPs in vitro and in vivo

A lipophilic dye DiL doped NPs (named DiL-loaded BA/Res@NP and DiL-loaded BA/Res@NP-PBP) were used to verify the in vitro uptake capacity of Colon-26 cells and Raw 264.7 cells. The cells were cultured in 6-well plates, and glass plates were placed in each well with a certain density. Cultured overnight to make the cells adhere to the wall and grow to the appropriate density. Added proper concentration of nanoparticles solution to cell culture medium and continue culture for 6–8 h. Then removed the medium, washed the cells twice with PBS and fixed the cells with 4% paraformaldehyde (PFA). The cells were washed twice more with PBS and phalloidin-FITC was added to stain the cytoskeleton. Washed the cells twice and added DAPI to stain the nuclei. Took out the cell glass plate and placed it on the slide to observe under confocal microscope.

To track NP in vivo after intravenous administration, DiR was encapsulated in BA/Res@NP as a fluorescent probe. Dextran sulfate sodium (DSS)-induced UC group were administered with BA/Res@NP or BA/Res@NP-PBP through intravenous injection. In vivo fluorescence imaging system (IVIS, Perkin Elmer; USA) was performed after 2 h intravenous injection. Then the mice were sacrificed. Collected their major organs (heart, liver, spleen, lung, and kidney) and colon tissues and imaged via IVIS. Histograms were used to reflect the fluorescence intensity of tissues and organs.

### In vitro anti-inflammatory activities of BA/Res@NP

Raw 264.7 cells were grown at the same cell concentration in a 6-well plate. After cell adherence, the medium was changed to a BA/Res@NP containing medium at a concentration of 200 µg/mL. After the BA/Res@NP were co-incubated with the cells for 6 h, removed the medium and washed the cells twice with PBS buffer. lipopolysaccharide (LPS) was used to induce inflammation in cells. Cells were collected after 4 h. The total RNA of the cells was extracted and reverse transcribed into cDNA, and the expression of pro-inflammatory cytokines in the cells was analyzed by real-time reverse transcription-polymerase chain reaction (RT-PCR) using SYBR Green/ROX qPCR Master Mix (Thermo Scientific), and the sequence of primers is listed in Additional file 1: Table S1. The experiment was repeated three times for each group.

### Animals

Female C57BL/6 mice (8-week-old) were purchased from the Medical Experimental Animal Center of Xi’an Jiaotong University, Shaanxi Province, China. They were fed in specific pathogen-free conditions with controlled ambient temperature and a 12 h of light and dark cycle. All the experiments involving mice were approved by the Institutional Animal Care and Use Committee (IACUC) of Xi’an Jiaotong University, Shaanxi Province, China (No. 2020-420). Groups of mice were randomly assigned.

### In vivo therapeutic effects of BA/Res@NP against acute and chronic colitis model

C57BL/6 mice were used in the acute UC model. Acute inflammation in the colon was induced by administering an aqueous solution containing 2.5% DSS to mice for 7 consecutive days. Groups of mice were randomly assigned. Each group was named control, DSS, Blank@NP, BA@NP group, Res@NP group, and BA/Res@NP group respectively. In experiment groups, mice were intravenously injected with Blank@NP, BA@NP, Res@NP, and BA/Res@NP (15 mg/kg) every 2 days. Observed and recorded the bodyweight, feces, and disease activity index daily. On the last day of the experiment, mice were sacrificed, and distal colons were collected for subsequent analysis.

Three cycles of DSS feeding are required to induce chronic UC model. After feeding 1.5% DSS solution for 7 days, replaced it with H_2_O for another 14 days. Repeated the above procedure for 3 cycles. The method of grouping and naming was the same as that of acute UC model. These mice were intravenously injected with Blank@NP, BA@NP, Res@NP, and BA/Res@NP (15 mg/kg) every 3 days. Feces were collected for assessment and the activity of MPO was measured. Observed and scored the disease activity (DAI) of mice daily. The scoring rules for the DAI are as follows: weight loss accounted for 4 points, fecal viscosity and fecal occult blood accounted for 3 points respectively [11].

### Biocompatibility assays

The biocompatibility of Blank@NP in vitro was determined by MTT assay. Raw 264.7 cells and Colon-26 cells were normally cultured in 96-well plates, and the wells at the edge of the plates were filled with PBS. Added different concentrations of Blank@NP (10, 20, 50, 100, 200, 400 µg/mL) to different wells for 24 and 48 h. All cell media were then removed and dimethyl sulfoxide (DMSO) was added to dissolve the resulting precipitate. Shaked the 96-well plate for 10 min to dissolve the precipitate completely. Finally, a microplate reader was used to measure the absorbance of each well at 570 nm. Set up the control group and zero adjustment group with at least 5 samples in each group.

To test the biocompatibility of Blank@NP in vivo, mice were recieved a 7 days intravenous injection of Blank@NP or control (saline). The disease activity including bodyweight and feces condition was recorded within 7 days. After the experiment, blood analysis and biochemical analysis were performed, and histopathological sections of major organs were observed [50, 51].

### Statistical analysis

All results were statistically analyzed by Student’s *t*-test and presented as the means ± standard deviations (SD). *p < 0.05, **p < 0.01, and ***p < 0.001 indicated statistical differences and NS represented no significance.

## Results

### Preparation and characterization of the PLGA-NP drug delivery system

Compared with other nano-drug delivery systems, PLGA, as FDA-approved polymers, have excellent biodegradability, strong ability to improve the solubility of hydrophobic drugs, and high encapsulation efficiency that effectively deliver more drugs to the site of disease and slow release of the encapsulated drug. Further, surface decoration of PLGA-PEG-Mal NPs is simple and can improve targeting efficiency; also, the PEG-Mal linker may increase circulation time in vivo and bioavailability of loaded drugs while protecting them from the immune response during circulation [52, 53].

**Fig. 2:**
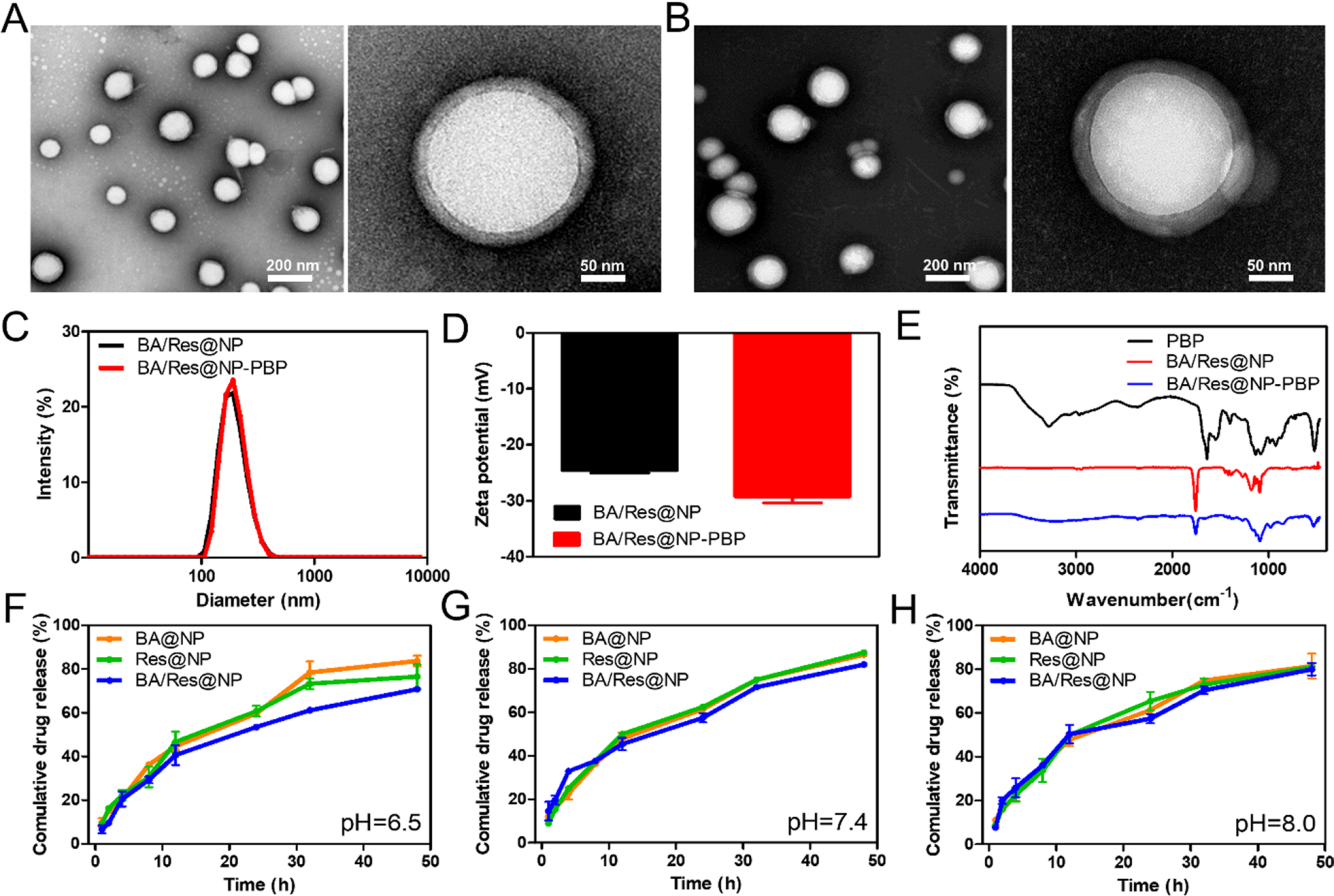
Characterization of NP.** A** The morphology of BA/Res@NP was characterized by TEM. **B** The morphology of BA/Res@NP-PBP. **C** Their size and **D** zeta potential. **E** FTIR spectra of PBP and BA/Res@NP-PBP showed the absorption of the PBP group at 988.68 cm^−1^, 928.98 cm^−1,^ and 524.82 cm^−1^. **F** Cumulative release profiles of BA and Res from BA@NP, Res@NP, and BA/Res@NP in pH 6.5, pH 7.4 (**G**), and pH 8.0 (**H**) were evaluated after different durations of dialysis (1, 2, 4, 8, 12, 24, 32 and 48 h), as quantified by ultraviolet spectrophotometer. FTIR: Fourier transform infrared spectroscopy. The experiments were repeated three times independently

To establish the PLGA-NPs and loaded them with BA and Res, we first optimized the composition for synthesizing BA-loaded NPs. Then we adjusted the ratio of Res to BA for incorporation. We fabricated five different formulations with a varied weight proportion of BA and Res. The formulation with the BA/Res proportion of 1:2 exhibited the highest BA/Res loading and encapsulation efficiencies. HPLC analysis revealed that the entrapment efficiencies of BA and Res in BA/Res@NP were 74.54% and 52.33%, and the loading capacities were 8.71% and 1.69%, respectively (Additional file 1: Table S2). TEM images showed that the morphology of both BA/Res@NP and BA/Res@NP-PBP were spherical, and the size distribution was narrow (Fig. 2A, B). Surface modification of PBP had no significant change in morphology. The particle sizes and zeta potentials were measured using the dynamic light scattering (DLS) method. The average size of BA/Res@NP was about 164.18 ± 0.8 nm, and zeta potential of − 25.46 ± 2.87 mV, while the respective measurements of BA/Res@NP-PBP were 184.3 ± 7.1 nm and − 28.02 ± 1.58 mV (Fig. 2C, D), indicating that after the surface decoration of the PBP, the physical characterization only slightly changed. In addition, BA/Res@NP-PBP had a narrow polydispersity index (PDI) of 0.051, suggesting that they are homogeneous in size, consistent with our TEM observations. Fourier transform infrared (FTIR) spectroscopy analysis also confirmed the successful coupling of PLGA-PEG-Mal and PBP (Fig. 2E).

We next investigated the cumulative release of BA and Res from the BA@NP, Res@NP, and BA/Res@NP in vitro. At different pH solutions, NPs showed similar drug release profiles (Fig. 2F–H), with ~ 60% of the encapsulated BA and Res releasing from the NPs at 24 h, and after 48 h the cumulative release reached ~ 80%. The drug release profiles indicated that BA and Res could be liberated from the hydrophobic core to yield a sustained drug release, avoiding sudden release.

### Evaluation of the NPs’ BRET-FRET effect

**Fig. 3:**
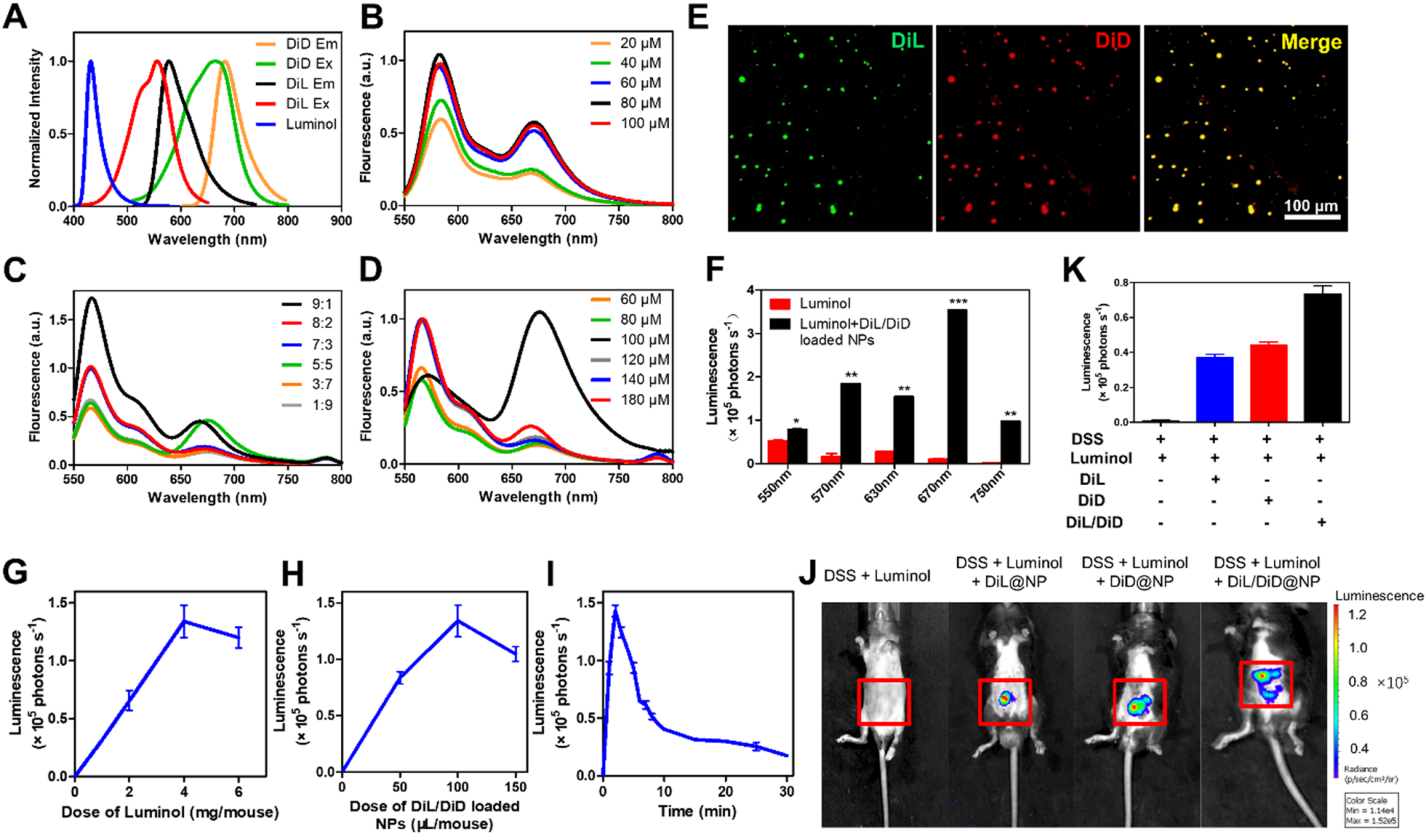
BRET-FRET progress was evaluated in vitro and in vivo. **A** The excitation and emission spectra of luminol, DiL, and DiD. **B** The fluorescence spectra of BA/Res@NP-PBP at different concentrations of DiL. **C** Fluorescence spectra of BA/Res@NP-PBP with different DiL and DiD proportions. **D** Fluorescence spectra of BA/Res@NP-PBP with different total concentration. **E** Images of DiL/DiD-loaded BA/Res@NP-PBP under a confocal microscope. **F** Histogram of bioluminescence intensity statistics under different excitation lights. **G** The changes of luminescence emission with the dose of luminol. **H** The changes of luminescence emission with the dose of DiL/DiD-loaded BA/Res@NP-PBP. **I** The changes of luminescence emission with the changes of the time point. **J** Bioluminescence images of mice from different treatment groups. **K** Histogram statistics of bioluminescence images. *p < 0.05, **p < 0.01, and ***p < 0.001

To build the FRET system, we chose two lipophilic dyes for an efficient energy transfer (Fig. 3A). DiL and DiD were selected to be incorporated in the hydrophobic inner cavity of PLGA-NPs (Fig. 1A). We first optimized the concentration of DiL in NPs to obtain the most robust fluorescence. We detected the fluorescence intensity of DiL at different concentrations. The results showed that DiL reached the highest fluorescence intensity at 80 µM, and the fluorescence intensity decreased with increasing concentration (Fig. 3B). After setting the concentration of DiL at 80 µM, we then mixed DiD to obtain different proportions of DiL/DiD mixture and detected their fluorescence intensity. The fluorescence intensity was the highest when the ratio of DiL to DiD was 9:1 (Fig. 3C). To get the highest energy transfer efficiency, we set the ratio at 9:1 and then adjusted the overall concentration of the DiL and DiD. The results showed that the fluorescence intensity increased with the concentration from 60 to 80 µM, and a strong fluorescence signal was observed at 670 nm (Fig. 3D). However, as the concentration increased, the fluorescence intensity decreased. Therefore, in the follow-up experiments, the concentration of DiL in the DiL/DiD-loaded BA/Res@NP-PBP was set as 80 µM and the ratio of DiL to DiD was 9:1. The final concentration was 100 µM to obtain the highest fluorescence intensity at 670 nm. Under a confocal microscope, we found that the DiL and DiD in NPs were overlapped together (Fig. 3E), indicating that DiL and DiD were encapsulated in NP and were very close to each other, which was a necessary condition between the donor and acceptor molecules for FRET (≤10 nm) [54, 55].

To further determine the BRET-FRET effect in a nonconjugated state between DiL/DiD-loaded BA/Res@NP-PBP and luminol, we established an in vitro MPO reaction with or without NPs. IVIS imaging system was used to detect luminescence. Large amounts of reactive oxygen species (ROS) and MPO were induced by adding phorbol myristate acetate (PMA) to the cell culture medium to generate photoemission of luminol. We detected a significantly stronger luminescence signal in the presence of luminol and NPs than luminol alone (Fig. 3F). Meanwhile, the signal was strong at 550 nm in the presence of luminol alone and much stronger at 670 nm in the presence of both luminol and NPs, indicating that the BRET-FRET effect between the luminol and DiL/DiD-loaded BA/Res@NP-PBP could extend the wavelength of the light emitted by luminol and MPO and red-shift to NIR region.

The MPO imaging capability of luminol and DiL/DiD-loaded BA/Res@NP-PBP NPs was further determined in the DSS-induced UC model, in which increased MPO activity presents in the inflammatory site of the colon. We improved the parameters of luminol and NPs bioluminescence imaging. The strongest luminescence signal was detected with the doses of luminol and NPs at 4 mg/mouse and 100 µL/mouse (Fig. 3G, H), respectively. Bioluminescence imaging was performed every 2 min after co-delivery of luminol (4 mg) and NPs (100 µL). And the strongest signal was obtained at 4 min after injection (Fig. 3I). Subsequently, the luminescence signal gradually decreased but could be detected until 30 min, providing sufficient time for MPO-dependent imaging. Also, injection of DiL/DiD-loaded BA/Res@NP-PBP and luminol together provided the peak bioluminescence signal with a nearly 24-fold increase (0.747 × 10^5^ versus 0.031 × 10^5^ photons/s) compared with luminol alone (Fig. 3J, K), suggesting an effective BRET-FRET effect between luminol and DiL/DiD-loaded BA/Res@NP-PBP in an MPO-dependent manner. Further, luminol and DiL or DiD loaded NPs resulted in only a 12-fold (0.389 × 10^5^ or 0.426 × 10^5^ versus 0.031 × 10^5^ photons/s, respectively) increase in luminescence emission (Fig. 3J, K). Therefore, the optimal BRET-FRET luminescence images were obtained after co-delivery of 4 mg luminol and 100 µL DiL/DiD-loaded BA/Res@NP-PBP NPs per mouse at 4 min, which can be used for the subsequent diagnosis and treatment of the UC model.

### Targeting ability of BA/Res@NP-PBP

To assess whether P-selectin could be used as a target for drug delivery in the inflamed area of the colon, we investigated P-selectin expressions in clinical samples from chronic colitis patients and associated the results with the progression of cancer using immunohistochemistry (IHC). As shown in Additional file 1: Fig. S1, the expression of P-selectin in healthy tissues was deficient but was much high in inflammatory colon sites and tumors at different stages. In addition, the expression level of P-selectin was positively correlated with the clinical stages of cancer (Additional file 1: Fig. S1), indicating that P-selectin could serve as a specific delivery target in UC therapy.

**Fig. 4:**
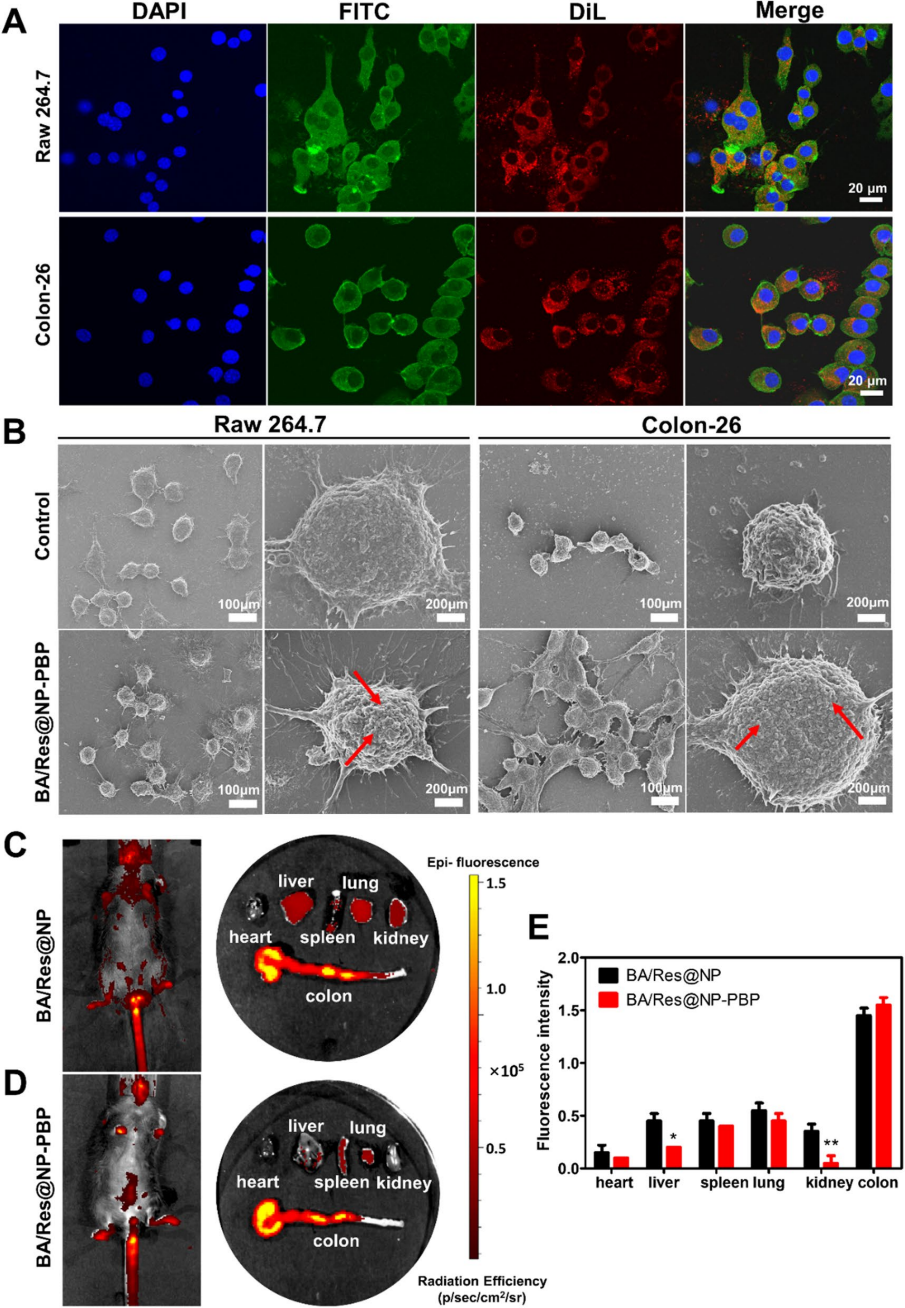
In vitro and in vivo targeting ability of BA/Res@NP-PBP.** A** Confocal images of cell uptake of NPs by Raw 264.7 cells and Colon-26 cells. The colors are as follows: DAPI, blue; FITC, green; DiL, red. Scale bar: 20 μm. **B** The binding of BA/Res@NP-PBP on Raw 264.7 cells and Colon-26 cells was observed by field emission scanning electron microscopy (FE-SEM). **C** Mice with DiR-labeled BA/Res@NP and **D** BA/Res@NP-PBP were imaged by in vivo imaging system (IVIS). **E** Histogram statistics were performed on fluorescence intensity of images (n = 5), *p < 0.05, **p < 0.01. The red arrows indicate NPs

The key for effective treatment of inflammation was to ensure that the drugs reach the inflammation site and are effectively internalized by the targeted cells. Raw 264.7 macrophage cells and Colon-26 epithelial-like are two major cells in colon-targeting drug delivery, which were chosen to evaluate the uptake efficiency of BA/Res@NP-PBP in vivo. DiL was encapsulated in BA/Res@NP-PBP as a fluorescent dye. After the DiL-labeled BA/Res@NP-PBP were incubated with Raw 264.7 and Colon-26 cells for 6 h, the localization of DiL-labeled BA/Res@NP-PBP in cells was observed by confocal microscope. Confocal imaging showed that after 6 h of incubation, a high DiL signal could be detected in the cells (Fig. 4A), indicating that the BA/Res@NP-PBP were successfully uptaken by Raw 264.7 cells and Colon-26 cells. However, compared with PBP modified BA/Res@NP (BA/Res@NP-PBP), the fluorescence signal of non-surface-functionalized NP (BA/Res@NP) in cells was weak (Additional file 1: Fig. S2), suggesting the limited target ability of BA/Res@NP and PBP modification can improve cell uptake efficiency. Furthermore, field-emission scanning electron microscopy (FE-SEM) was utilized to visualize the real-time binding of BA/Res@NP-PBP with Raw 264.7 cells and Colon-26 cells. The versatility of FE-SEM allowed us to obtain a detailed characterization of NPs’ processes of adsorption and endocytosis. FE-SEM images revealed that large amounts of BA/Res@NP-PBP (marked by red arrow) were present on the surface of Raw 264.7 cells and Colon-26 cells (Fig. 4B), indicating PBP-medicated active-targeting could significantly improve the binding ability between NPs and cells.

Next, we next studied the enhanced targeting effect of BA/Res@NP-PBP to the inflamed colon compared to BA/Res@NP in mice. DSS-induced colitis mice were intravenously injected with DiR-loaded BA/Res@NP-PBP or DiR-loaded BA/Res@NP. As a lipophilic NIR fluorescent dye, DiR is the best dye for in vivo imaging because of its deep penetration depth, which makes deep tissue imaging possible. After 12 h administration, collected the major organs (including the colon) in each group, obtained the in vivo imaging by IVIS. In fact, mice with colitis received the DiR loaded BA/Res@NP-PBP and showed significantly stronger fluorescent signals in the colon than the DiR loaded BA/Res@NP treated groups (Fig. 4C, D) validating a significantly improved colon-targeting effect. Additionally, fluorescence was also observed in the main organs from both groups (Fig. 4C, D). In contrast, in the DiR-loaded BA/Res@NP-PBP group, we found significantly reduced accumulation of NPs in the liver and kidney, suggesting PBP modification avoids potential NP systemic biodistribution and toxicity. Quantitative DiR fluorescent intensity from the colon and other organs was analyzed by region of interest (ROI) and represented by a histogram, as shown in Fig. 4E. Both BA/Res@NP-PBP and BA/Res@NP target the inflamed colon in the colitis mice, with the former showing slightly higher targeting ability.

### Synergistic anti-inflammatory effects of BA/Res@NP in vitro

**Fig. 5:**
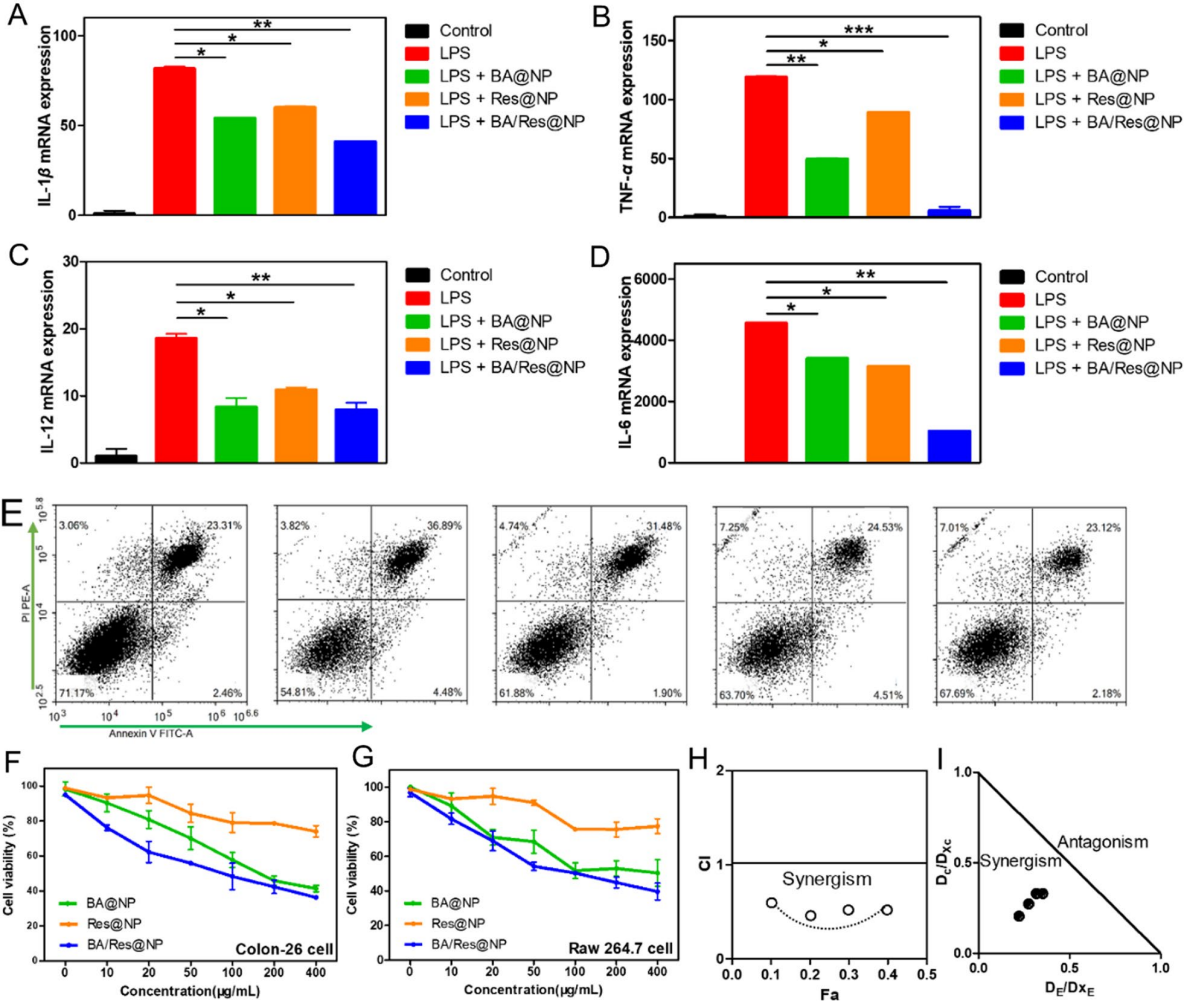
The anti-inflammatory effect of BA/Res@NP and synergistic effects of BA and Res.** A** mRNA expression levels of IL-1*β*, TNF-*α* (**B**), IL-12 (**C**), and IL-6 (**D**) in LPS-induced Raw 264.7 cells were quantified by qRT-PCR (n = 5). **E** Cell apoptosis was detected by flow cytometry in Control, LPS, LPS + BA@NP, LPS + Res@NP, and LPS + BA/Res@NP group, respectively. **F **In vitro cytotoxicity of NPs in Colon-26 cells. **G** In vitro cytotoxicity of NPs in Raw 264.7 cells. **H** CI-Fa plot. **I** Isobologram. *p < 0.05, **p < 0.005, ***p < 0.001

 The anti-inflammatory effect of BA/Res@NP was demonstrated in a model of LPS-induced inflammation in macrophages. After LPS induction, the level of pro-inflammatory cytokines (IL-1*β*, IL-6, TNF-*α*, and IL-12) in macrophages increased rapidly, which was significantly higher than the expression of the negative control group (Fig. 5A–D). Strikingly, pretreatment of BA@NP or Res@NP could effectively reduce the expression levels of pro-inflammatory factors, suggesting that BA and Res had significant anti-inflammatory activity, which was consistent with the previous reports. Moreover, BA/Res@NP down-regulated the expression levels of pro-inflammatory cytokines most, compared to BA@NP alone and Res@NP alone groups, suggesting a potential synergistic effect between BA and Res.

LPS-induced inflammation of Raw 264.7 cells inevitably led to cell death. The percentage of apoptotic was revealed by Annexin V-FITC/PI assays. The results showed the proportion of apoptotic cells in LPS group was the highest (36.89 ± 7.4%), BA@NP and Res@NP group could reduce the number of apoptotic cells (31.48 ± 9.2%, 24.53 ± 2.1%), but the proportion of apoptotic cells in the synergistic administration group was the lowest (23.12 ± 2.1%) (Fig. 5E), demonstrating anti-inflammatory effects of BA and Res, and the combination of the two drugs was better. The results were presented by flow cytometry.

To confirm the synergistic effect of BA and Res, we also compared the effects of BA@NP alone, Res@NP alone, and BA/Res@NP combination treatments on cell viability using MTT assays. At a certain concentration, both BA and Res could reduce the cell viability in a dose-dependent manner (Fig. 5F, G). The Chou-Talalay method was a widely accepted method for quantitative analysis of drug synergies. Finally, the combination index (CI) could describe the additive, synergistic and antagonistic effects of drugs. The results were denoted by the fraction affected (Fa) plot and the isobologram, respectively. So the cell viability with the CI values of 0.60, 0.43, 0.52, and 0.51 for the BA/Res@NP concentration decreasing 10% of cell viability IC_10_, IC_20_, IC_30_, and IC_40_, respectively, reflecting a significant synergistic effect between BA and Res. In addition, the CI values versus Fa plot (Fig. 5H) and the isobologram (Fig. 5I) confirmed that BA and Res have a synergistic therapeutic effect indeed.

### Biocompatibility of NP in vitro and in vivo

Biocompatibility is a key factor to consider when evaluating a novel nano delivery platform. The biocompatibility of Blank@NP on Colon-26 cells in vitro was investigated by MTT assay. The Colon-26 cells were treated with Blank@NP at the different tested concentrations (up to 400 µg/mL). After 24 h of co-culture, the cell viability was not significantly affected (Additional file 1: Fig. S3A). When the incubation time was extended to 48 h, other conditions remained unchanged, the cell viability was not significantly affected either (Additional file 1: Fig. S3B), indicating this nano delivery system had no toxicity to cells.

Then we also evaluated the biocompatibility of Blank@NP in vivo. Blank@NP was injected intravenously for a week and measured the bodyweight change daily. The results showed that there was no significant change in weight loss compared with the control group (Additional file 1: Fig. S4). Tissue sections stained with H&E showed that there was no damage to the main organs in the Blank@NP group, which was consistent with the control group (Additional file 1: Fig. S5). The structure of the endocardium, myocardial membrane, and epicardium of heart tissue are apparent in both the Blank@NP group and the control group. Hepatocytes and hepatic lobules were intact, and no fibrosis was found in lung specimens. No abnormality was found in blood analysis and biochemistry examination (Additional file 1: Fig. S6). The liver and kidney injury indicators were also within the normal range in both the Blank@NP and control groups. The in vitro and in vivo results indicated that Blank@NP were with good biocompatibility and non-toxic, which could be served as a safe nanocarrier for drug delivery.

### Administration of BA/Res@NP attenuates acute colitis

**Fig. 6:**
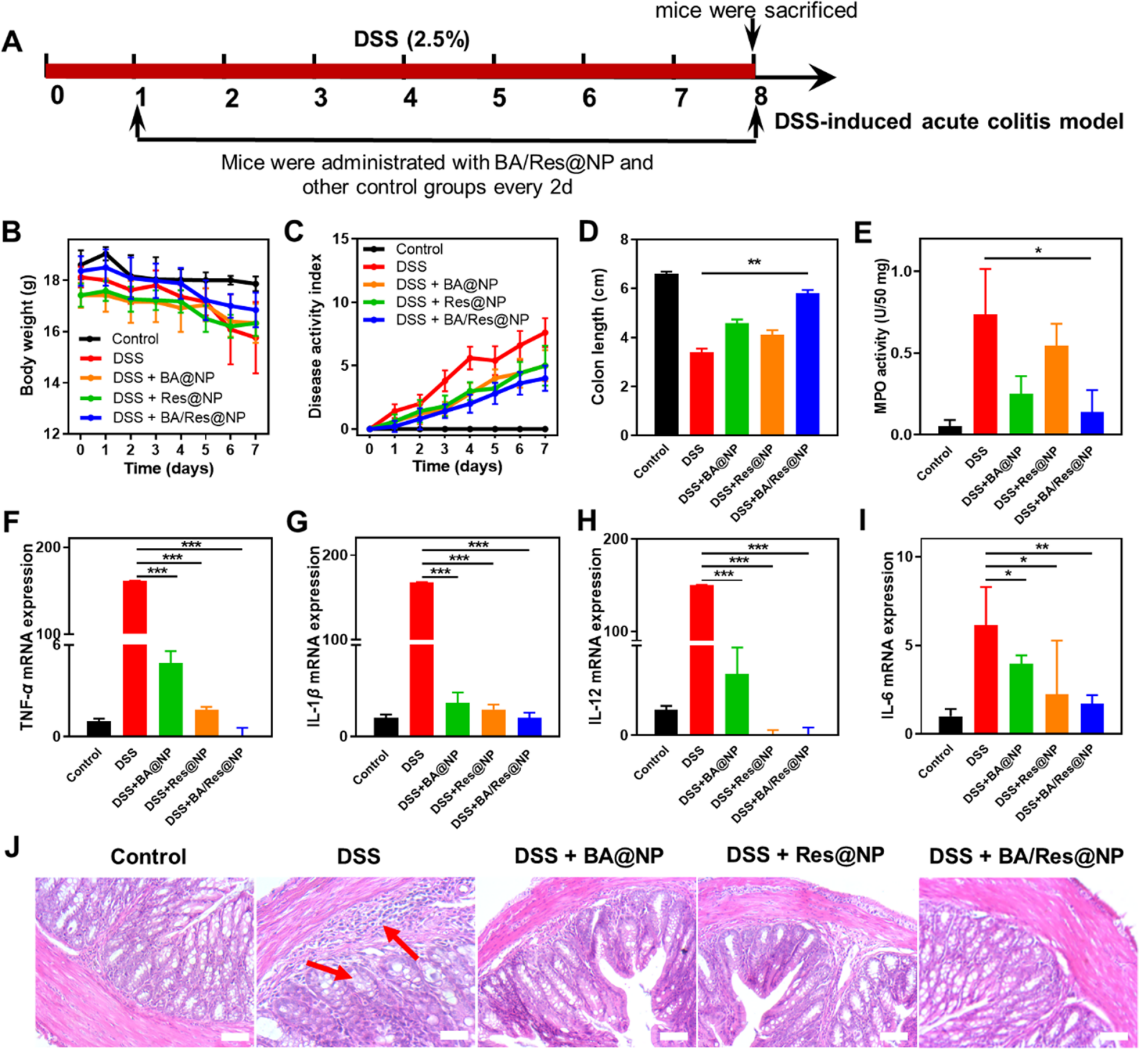
The therapeutic outcomes of BA/Res@NP against acute UC.** A** Methods of modeling acute UC. **B** Weight changes over time. **C** Disease activity index. **D** Colon length. **E** MPO activities. **F**–**I** mRNA expressions of TNF-*α*, IL-1*β*, IL-12, and IL-6 were evaluated by rtPCR (n = 5). **J** H&E-stained sections of colon tissues in different groups. Scale bar: 200 μm. Arrows: inflammatory cells infiltration. *p < 0.05, **p < 0.005, ***p < 0.001

To evaluate the anti-inflammatory effect of BA and Res in vivo, we first investigated whether BA@NP, Res@NP, and BA/Res@NP could relieve DSS-induced acute colitis. DSS-induced acute colitis in mice is a model with high similarity to human colitis, and the specific implementation method is shown in Fig. 6A. During the treatment, we observed that BA@NP, Res@NP, and BA/Res@NP significantly reduced the body weight loss and lowered the DAI within 7 days compared to the DSS group (Fig. 6B, C), indicating reduced inflammation DAI with NPs treatment. On the 8th day of the experiment, the mice were sacrificed, and the colon tissues were obtained. By measuring the length of the colons, the DSS group was the shortest, while BA@NP, Res@NP, and BA/Res@NP groups were longer than the DSS group (Fig. 6D, Additional file 1: Fig. S7). In addition, we measured colonic MPO activities, a factor that reflected inflammation. DSS treatment significantly boosted the MPO activity but was notably decreased by BA@NP, Res@NP, and BA/Res@NP, with the BA/Res@NP combinational therapy, had the best effect on reducing the MPO activity (Fig. 6E).

We then examined the expression level of pro-inflammatory factors in colon tissues of different groups of mice. The results showed that BA@NP, Res@NP, and BA/Res@NP significantly decreased the mRNA level of TNF-*α*, IL-1*β*, IL-12, and IL-6 (Fig. 6F–I), suggesting that BA@NP, Res@NP, and BA/Res@NP could reduce the transcription of pro-inflammatory factors and effectively alleviated DSS-induced acute colitis.

Statistical analysis of histological scores of BA@NP, Res@NP, and BA/Res@NP on acute UC was performed by H&E-stained sections. DSS-induced but untreated mice exhibited significant signs of inflammation, including ulceration, goblet cell decreased, crypt disappeared, mucosal thickening, and lymph node formation (Fig. 6J). In contrast, treatment with BA@NP, Res@NP, or BA/Res@NP attenuated these inflammatory manifestations, particularly in the context of local lymphocytic infiltration. BA/Res@NP group presented the best efficacy reflected by the histological score levels (Additional file 1: Fig. S8). These findings indicate that BA and Res exert excellent anti-inflammatory effects and combinational treatment results in the best attenuation for colonic inflammation in the DSS-induced UC.

### Impact of BA/Res@NP on gut microbiota

**Fig. 7:**
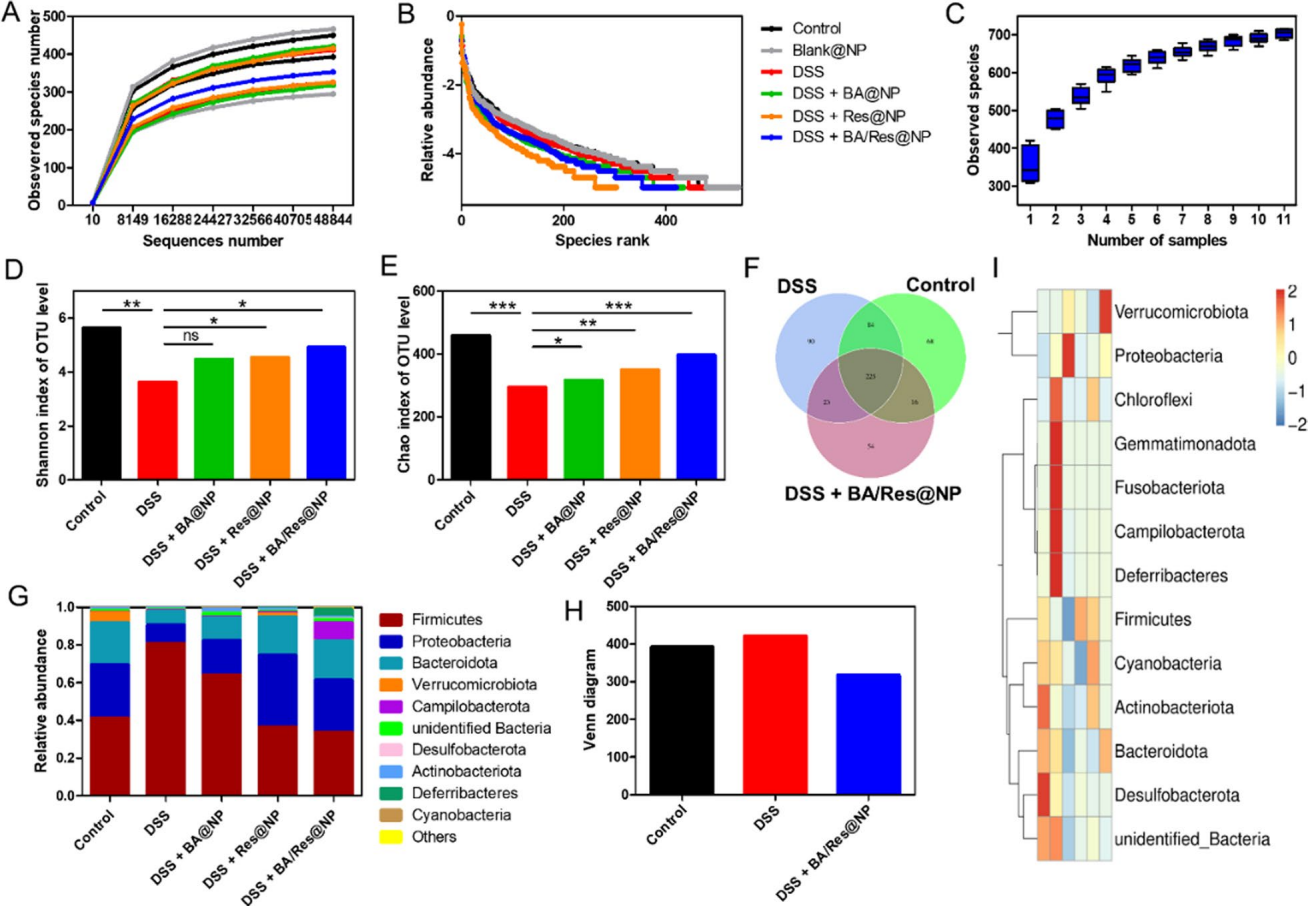
The effect of BA/Res@NP on gut microbiota.** A** Rarefaction curve. **B** Rank-abundance curve. **C **Species accumulation boxplot of different mice groups. **D** Shannon. **E** Chao index. **F** Venn diagram. **G** Histogram of colony composition. **H** Statistical histogram of a Venn diagram. **I** Heatmap of gut microbiota in different groups

Studies have shown a strong correlation between intestinal diseases and its gut microbiota. Gut microbiota was an important part of the intestinal environment, and the normal gut microbiota maintained a relatively stable state. Gut microbiota could promote the operation of nutrients in the intestinal tract, preserve the healthy homeostasis of the intestine, and promote the development of the immune system. A large number of studies have confirmed that the microbiota of UC patients is significantly different from that of healthy people, which due to the changes in the occurrence and development of UC [56–58]. To determine whether generated BA/Res@NPs could maintain gut microbiota homeostasis, 16S rRNA sequencing analysis was used to investigate the gut microbiota from collected fecal samples. The method validation study showed that with the increasing sample size, the curves representing species diversity and richness gradually flattened (Fig. 7A, B), indicating that the sample size was enough for sequencing. The rank abundance curve also represented the richness and evenness of samples (Fig. 7C). These results showed that the results analyzed by 16S rRNA sequencing were reliable.

The beta diversity (number of species) in the healthy control mice was higher than other groups. NP-treated groups showed significantly increased beta diversity (Shannon index of operational taxonomic units [OTUs]) that was reduced by DSS-only treatment (Fig. 7D). A similar result was reflected by the Chao index of OTUs (Fig. 7E). Moreover, there was a positive correlation between the diversity of gut microbiota and the effect of NPs on UC treatment, indicating that the more microbiota, the better the therapeutic effect of UC. As shown in the Venn diagram (Fig. 7F, H), certain bacterial species appeared in all three groups, indicating these microbiotas might contribute to maintaining the homeostasis of the gut environment. Data presented in Fig. 7G showed that the majority of the dominant bacteria in the feces of the healthy control group were symbiotic bacteria, such as *Firmicutes*, *Proteobacteria*, and *Bacteroidota*. On the contrary, the DSS group showed a significant reduction in the diversity of symbiotic bacteria. While, the variety of symbiotic bacteria in NP treatment groups increased within the dominant species, in which BA/Res@NP had the most increased diversity. These change trends of predominant bacteria were consistent with the treatment results of UC. Moreover, heatmap of the gut microbiota composition in different treatment groups represented that the more similar the composition was to that of the healthy control group, the better the therapeutic effect was generated of UC (Fig. 7I). These results demonstrated that the imbalance of intestinal microbiota is closely related to the inflammation in the gastrointestinal tract. Moreover, BA/Res@NP could modulate the imbalanced gut microbiota ratio towards a near-healthy ratio, similar to the effect of BA/Res@NP in treating acute UC.

### Administration of BA/Res@NP attenuates chronic colitis

**Fig. 8:**
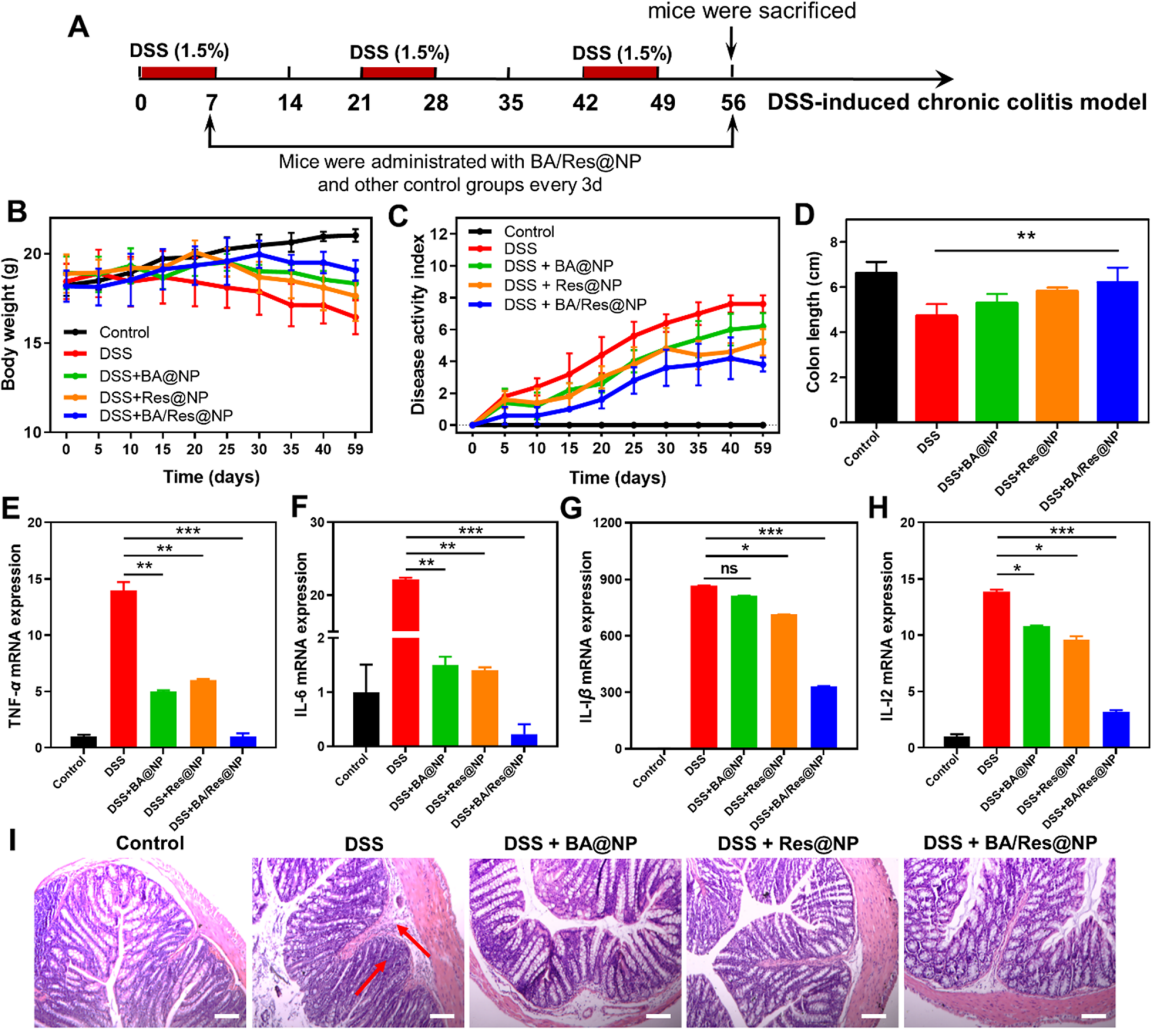
The therapeutic outcomes of BA/Res@NP against chronic UC. **A** Methods of modeling chronic UC. **B** Time-dependent variations of body weights. **C** Disease activity index. **D** Colon length. **E**–**H** The levels of mRNA expression of pro-inflammatory mediators (n = 5). **I** Histological H&E staining sections of the colons. Scale bar: 200 μm. Arrows: inflammatory cells infiltration. *p < 0.05, **p < 0.005, ***p < 0.001

We also established a chronic UC model induced by DSS, which could well reflect the relapsing and long-lasting of IBDs in humans so as to evaluate further the anti-inflammatory effect of BA@NP, Res@NP, and BA/Res@NP. The modeling method is shown in Fig. 8A. After three rounds of DSS feeding, the weight of mice decreased significantly in the DSS group. Mice treated with each NP showed slight bodyweight loss, DAI, and increased colon length (Fig. 8B–D). Results further showed that treatment with BA@NP, Res@NP, or BA/Res@NP significantly decreased the expression of colonic pro-inflammatory cytokines, such as TNF-*α*, IL-1*β*, IL-6, and IL-12 (Fig. 8E–H). Anti-inflammatory effects of BA@NP and Res@NP were further verified by histological analysis of H&E-stained sections, including reduced neutrophil infiltration and disappeared ulcers (Fig. 8I). Overall, the performance of BA@NP and Res@NP in the chronic colitis model was consistent with that in acute UC, and the BA/Res@NP combined with two drugs had a synergistic effect.

## Discussion

This study aims to construct a biocompatible theranostic NP platform to detect the severity of the colitis and deliver drugs to the colitic region to treat the UC. The NP platform was constructed using PLGA polymer, which had the advantages of biodegradable, biocompatible, and controllable drug release properties. This NP system also allows the encapsulation of multiple drugs; as such, two drug candidates (BA and Res) were encapsulated in the biodegradable PLGA core. The outer layer was decorated with PBP and was linked to the PLGA core via PEG-Mal; PEG-Mal linker might improve encapsulation efficiency, reduce immunogenicity, and prolong the circulation time in vivo. PBP-functionalized PLGA-PEG-Mal NPs also exhibited excellent biocompatibility and targeting ability and increased the drug accumulation at the colitic site, indicating this PLGA-NP-PBP system is a safe and effective drug carrier.

Anti-inflammation therapy is often associated with antibiotic abuse and gastrointestinal severe side effects. As natural compounds, BA and Res have potent anti-inflammation activity and are low cytotoxicity. Studies have shown that oral administration of Res can reduce colonic injury in mice with colitis [59, 60]. Clinical research showed that it is safe to use Res as a dietary supplement in IBD patients and effectively reduce disease symptoms [31]. Although the potential mechanisms of the Res’s protective role in the intestine are unclear, administration of BA/Res@NP achieved a comparable efficacy to BA or Res alone, even at a tenfold lower dose. This finding suggested that NP delivery could effectively increase the effectiveness or reduce the side effects of BA and Res. Moreover, BA/Res@NP was administered in advance of inflammation in both in vivo and in vitro models, meaning that BA/Res@NP might have a prophylactic effect on UC. Further, the protective effect of BA/Res on the colon has similar efficacy in humans. However, the safety and effectiveness of BA/Res@NPs for human consumption have to undergo rigorous clinical trials.

Hydrophobic drugs suffered from poor pharmacokinetic (PK) profiles, such as low absorption, low bioavailability, and fast clearance from the biosystem [61]. Using the NP described as mentioned as a nano-drug delivery system could be a feasible method to improve the PK profile of loaded BA and Res. NP encapsulation can also avoid the instant and premature release of drugs, and the cumulative release 48 h can reach more than 80%. Drug-enriched PLGA core delayed the release of the BA and Res from NPs, which represented a sustained therapeutic effect. We then observed that Res was released from PLGA-NPs slightly faster than BA; this is probably because Res’s molecular weight is much lower than BA. In addition, in our work, PLGA is a stable polymer maintaining slow-release characteristics in the solution of different pH values (pH = 6.5, 7.4, 8.0). With the increase of time, the ester bond on PLGA broke, resulting in various degrees of hydrolysis so that the encapsulated drug slowly released, avoiding a sudden release. However, hydrolysis increased under acidic conditions, thus increasing the drug release [62]. Moreover, the pH value of the blood is about 7.4. pH 6.5 and 8.0 were weakly acidic and weakly alkaline, respectively, so the influence of different pH values on drug release in our work was not apparent, further demonstrating the good stability of PLGA as a delivery carrier.

In the treatment of inflammatory diseases, the abuse of anti-inflammatory drugs has brought serious side effects. In clinical practice, systemic exposure can be reduced by combining drugs. But some drug combinations are simply additive rather than synergistic. Chou-Talalay methods describe the drug combination effect qualitatively and quantitatively by statistical analysis of the dose-effect relationship obtained from MTT assay. We assessed the in vitro synergistic effect of BA and Res on Colon-26 cells and Raw 264.7 cells using the Combination index (CI) theorem of the Chou-Talalay Method. CI is the most direct indicator of drug combinations [63], which reflects the correlation between BA and Res. CI < 1 means synergism, CI > 1 suggests antagonism, and CI = 1 indicates additive, respectively [64]. MTT assay was carried out to evaluate the BA/Res@NP with a BA to Res ratio of 1:1, 2:1, 3:1, 1:2, 1:3 (w/w) to obtain the CI of each group. There is a synergistic effect between the two drugs when the weight ratios of BA to Res were 1:1, 1:2, 2:1, and 1:3. Among them, the weight ratio of 1:2 showed the strongest synergism, indicating that it is an optimum BA to Res ratio in the NPs formulations.

Real-time, non-invasive luminescence imaging is widely used in detecting, diagnosing, and monitoring various diseases [65, 66], but it is not much in inflammatory conditions, especially IBD. Moreover, there are few safe and sensitive in vivo imaging agents. It has been reported that FRET pairs were encapsulated into nano delivery carriers or conjugated FRET pairs with polymers as nanoprobes for imaging [67, 68]. This novel type of probe can visualize drug nanocarriers’ in vivo transportation biodistribution and monitor their stability and integrity via non-invasive real-time imaging [69]. Here, we loaded fluorescent dyes DiL and DiD into BA/Res@NP and injected them together with the small-molecule luminescent probe luminol, employing the BRET-FRET effect between luminol and NP to transfer the light emitted by luminol to the NIR region. The luminescence of DiL/DiD-BA/Res@NP is positively correlated with MPO level, which is a pro-inflammatory mediator commonly elevated in both acute and chronic colitis. In UC mice models of inflammatory diseases, co-delivery of DiL/DiD-BA/Res@NP and luminol showed significantly higher sensitivity than luminol alone. This is because free luminol is a small molecule, which is quickly cleared by the biosystem, leading to low probe efficiency and poor imaging quality [70]. Co-delivered luminol markedly enhanced DiL/DiD-loaded BA/Res@NP luminescent signal via BRET-FRET, which should be attributed to the high vascular permeability at the colon tissue, making the colon-targeting of DiL/DiD-loaded BA/Res@NP stronger. Luminol detected MPO at the inflammatory site and transferred the emitted light to highly aggregated DiL/DiD-loaded BA/Res@NP, effectively ensuring the efficiency of BRET-FRET between luminol and NP. Furthermore, engineered DiL/DiD-BA/Res@NP as a sensitive, safe, and reliable probe have great potential for monitoring the process of colitis and other deep tissue inflammation.

## Conclusions

In summary, we developed a P-selectin targeting PLGA-NP delivery system to co-deliver natural compounds BA and Res to the inflammatory site of the colon. Meanwhile, the integrated BRET-FRET energy transfer relay could red-shift the light emitted by luminol to NIR region, making MPO-dependent imaging of deep tissues possible. The formulated BA/Res@NP-PBP NPs exhibited nano-size, narrow size distribution, and negative zeta potential with sustained release profile in vitro. In addition, the anti-inflammatory effects of individual BA or Res were effectively improved by the polymer encapsulated co-delivery system, and the targeting effect mediated by P-selectin increased the accumulation of NPs in the inflammatory colon. This methodology served as a potential co-delivery natural nano-drug delivery system for UC-targeting therapy and provided a non-invasive and highly sensitive approach for accurately visualizing inflammatory diseases.

## Supplementary Information

**Additional file 1: Fig. S1.** P-selectin expressions in clinical samples from chronic colitis and adenocarcinoma patients. A human P-selectin antibody was used to observe the expression level of P-selectin in inflammatory site of colon and cancer tissues via a human tissue microarray counterstained with hematoxylin. Scale bar: 20 μm. **Fig. S2.** Cellular uptake of BA/Res@NP and BA/Res@NP-PBP by colon-26 cells (A) and Raw 264.7 cells (B). The cell nucleus was stained by DAPI (Blue), cytoskeleton was stained by FITC-phalloidin (Green), and NPs were labeled by lipophilic carbocyanine dye, DiL (Red), scale bar: 20 μm. **Fig. S3.** In vitro biocompatibility of Blank@NPs. Cell vitablity of Colon-26 cells after being incubated with Blank@NPs compared with Control at varied concentrations (0, 10, 20, 50, 100, 200, and 400 μg/ mL) for 24 h (A) and 48 h (B). **Fig. S4.** Changes of body weight over time in Control group and Blank@NP-treated group. **Fig. S5.** In vivo toxicity was evaluated by H&E staining of vital organ tissues. The organs were harvested, fixed in 10% formalin, embedded in paraffin, sectioned, subjected to H&E staining, and examined for histological assessment. Representative images are shown (n=3). Scale bar: 50 µm. **Fig. S6.** Partial blood test (A) and biochemical parameters (B) of mice in the Control group and Blank@NP-treated group. Each point represents the mean ± SEM (n=3). **Fig. S7.** Colon length in different groups of mice. **Fig. S8.** Histopathologic score in different groups of mice. **Table S1.** Primers used for Real-time PCR. **Table S2.** The entrapment efficiency and loading capacity of NPs doped with different BA/Res ratios. **Experiment methods:** Staining of colon microarrays; Cell apoptosis study; In vivo monitoring of inflammation during acute UC; Histological analyses of tissue sections by hematoxylin and eosin staining; Impact of BA/Res@NP on intestinal microbiota.

## Data Availability

The data underlying this article will be shared on reasonable request to the corresponding author.
