# Supplementary material for: All-in-one theranostic nano-platform based on polymer nanoparticles for BRET/FRET-initiated bioluminescence imaging and synergistically anti-inflammatory therapy for ulcerative colitis
Source: J Nanobiotechnology. 2022 Mar 2;20:99. doi: 10.1186/s12951-022-01299-8 (PMC8889649; doi:10.1186/s12951-022-01299-8)
Supplement: Supplementary file 1 — Additional file 1: Fig. S1. P-selectin expressions in clinical samples from chronic colitis and adenocarcinoma patients. A human P-selectin antibody was used to observe the expression level of P-selectin in inflammatory site of colon and cancer tissues via a human tissue microarray counterstained with hematoxylin. Scale bar: 20 μm. Fig. S2. Cellular uptake of BA/Res@NP and BA/Res@NP-PBP by colon-26 cells (A) and Raw 264.7 cells (B). The cell nucleus was stained by DAPI (Blue), cytoskeleton was stained by FITC-phalloidin (Green), and NPs were labeled by lipophilic carbocyanine dye, DiL (Red), scale bar: 20 μm. Fig. S3. In vitro biocompatibility of Blank@NPs. Cell vitablity of Colon-26 cells after being incubated with Blank@NPs compared with Control at varied concentrations (0, 10, 20, 50, 100, 200, and 400 μg/ mL) for 24 h (A) and 48 h (B). Fig. S4. Changes of body weight over time in Control group and Blank@NP-treated group. Fig. S5. In vivo toxicity was evaluated by H&E staining of vital organ tissues. The organs were harvested, fixed in 10% formalin, embedded in paraffin, sectioned, subjected to H&E staining, and examined for histological assessment. Representative images are shown (n=3). Scale bar: 50 µm. Fig. S6. Partial blood test (A) and biochemical parameters (B) of mice in the Control group and Blank@NP-treated group. Each point represents the mean ± SEM (n=3). Fig. S7. Colon length in different groups of mice. Fig. S8. Histopathologic score in different groups of mice. Table S1. Primers used for Real-time PCR. Table S2. The entrapment efficiency and loading capacity of NPs doped with different BA/Res ratios. Experiment methods: Staining of colon microarrays; Cell apoptosis study; In vivo monitoring of inflammation during acute UC; Histological analyses of tissue sections by hematoxylin and eosin staining; Impact of BA/Res@NP on intestinal microbiota. [file 12951_2022_1299_MOESM1_ESM.docx]

**All-in-one theranostic nano-platform based on** **polymer nanoparticles for** **BRET/FRET-initiated bioluminescence imaging and synergistically anti-inflammatory therapy for ulcerative colitis**

Xiangji Yan^1,2^, Chunhua Yang^3^, Mei Yang^1,2^, Yana Ma^1,2^, Yuanyuan Zhang^1,2^, Yujie Zhang^1,2^, Cui Liu^1,2^, Qiuran Xu^4,*^, Kangsheng Tu^5,*^, and Mingzhen Zhang^1,2,*^

**Affiliations:**

1. School of Basic Medical Sciences, Xi’an Key Laboratory of Immune Related Diseases, Xi’an Jiaotong University, Xi’an, Shaanxi, 710061, China
2. Key Laboratory of Environment and Genes Related to Diseases, Xi’an Jiaotong University, Ministry of Education, Xi’an, Shaanxi, 710061, China
3. Institute for Biomedical Sciences, Center for Diagnostics and Therapeutics, Digestive Disease Research Group, Georgia State University, Atlanta, Georgia, 30302, United States.
4. Laboratory of Tumor Molecular Diagnosis and Individualized Medicine of Zhejiang Province, Zhejiang Provincial People’s Hospital, Affiliated People’s Hospital, Hangzhou Medical College, Hangzhou, Zhejiang, 310014, China
5. Department of Hepatobiliary Surgery, the First Affiliated Hospital of Xi’an Jiaotong University, Xi’an, Shaanxi, 710061, China

* Authors for correspondence

Email: windway626@sina.com (Q, Xu); tks0912@foxmail.com (K, Tu); mzhang21@xjtu.edu.cn (M, Zhang)

**Additional file 1**


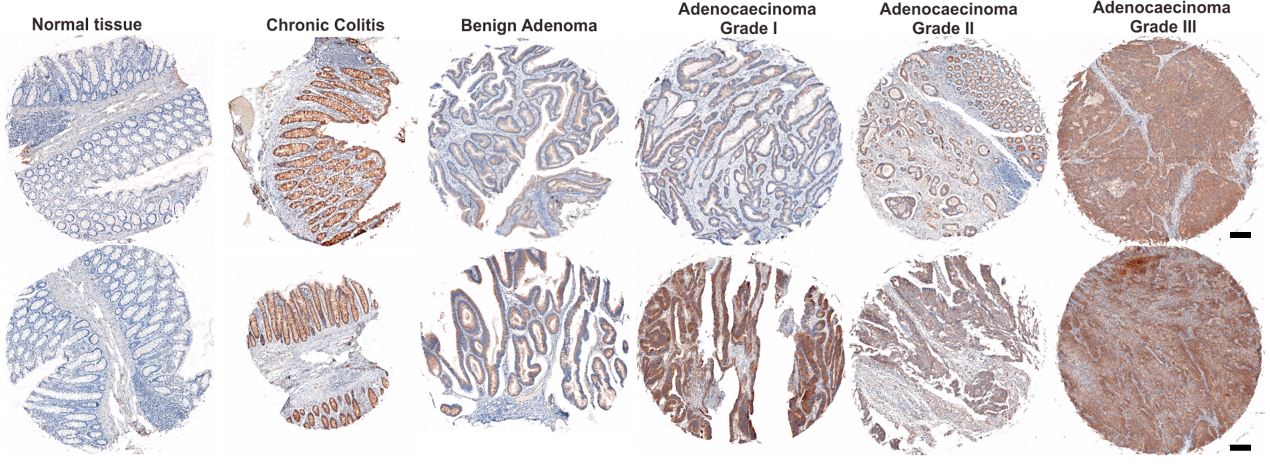


**Figure S1.** **P-selectin expressions in clinical samples from chronic colitis and adenocarcinoma patients.** A human P-selectin antibody was used to observe the expression level of P-selectin in inflammatory site of colon and cancer tissues via a human tissue microarray counterstained with hematoxylin. Scale bar: 20 μm.


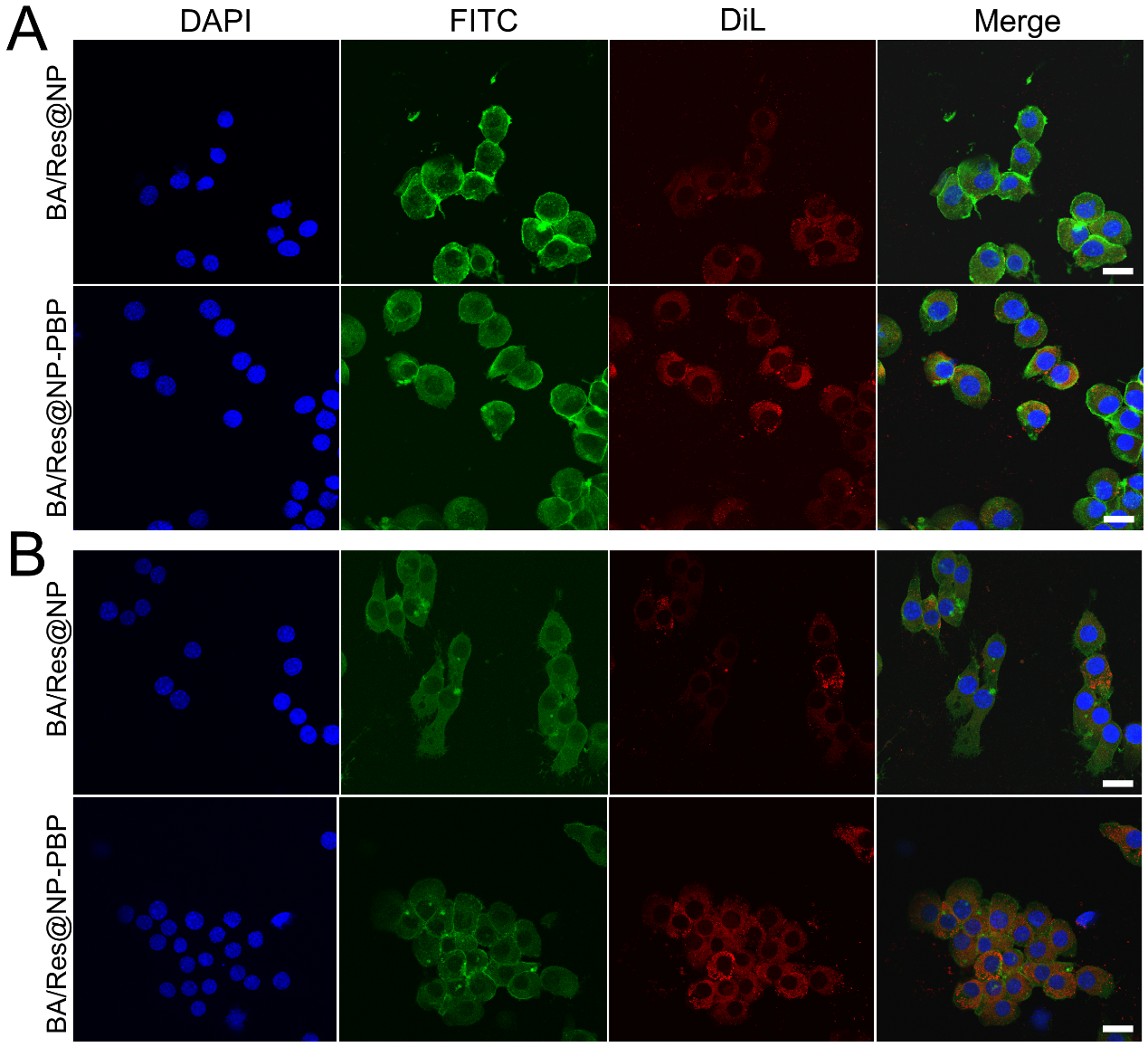


**Figure S2.** **Cellular uptake of BA/Res@NP and BA/Res@NP-PBP by colon-26 cells (A) and Raw 264.7 cells (B)*.*** The cell nucleus was stained by DAPI (Blue), cytoskeleton was stained by FITC-phalloidin (Green), and NPs were labeled by lipophilic carbocyanine dye, DiL (Red), scale bar: 20 μm.


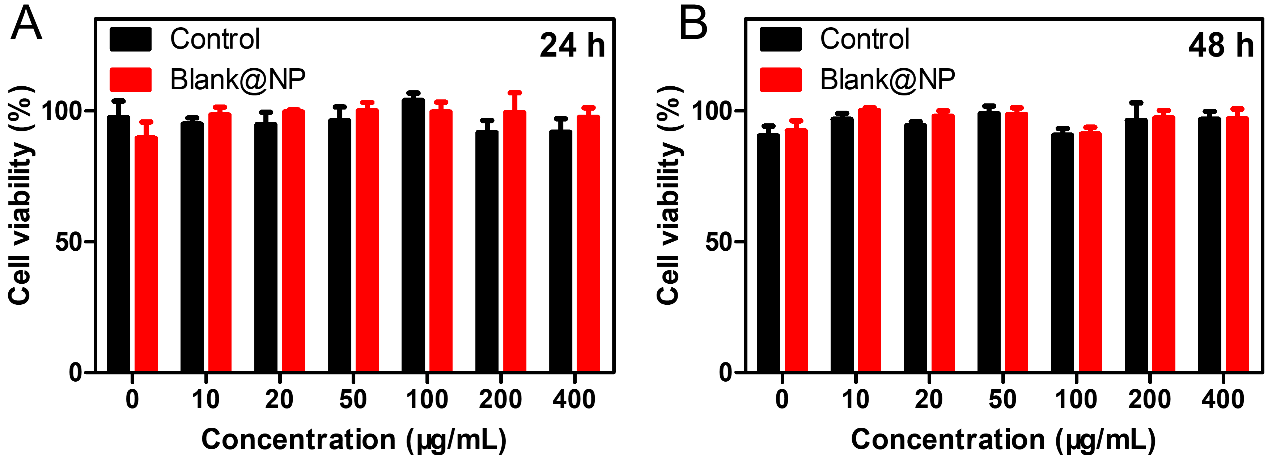


**Figure S3. *In vitro* biocompatibility of Blank@NPs.** (A) Cell vitablity of Colon-26 cells after being incubated with Blank@NPs compared with Control at varied concentrations (0, 10, 20, 50, 100, 200, and 400 μg/ mL) for 24 h and 48 h (B).

**
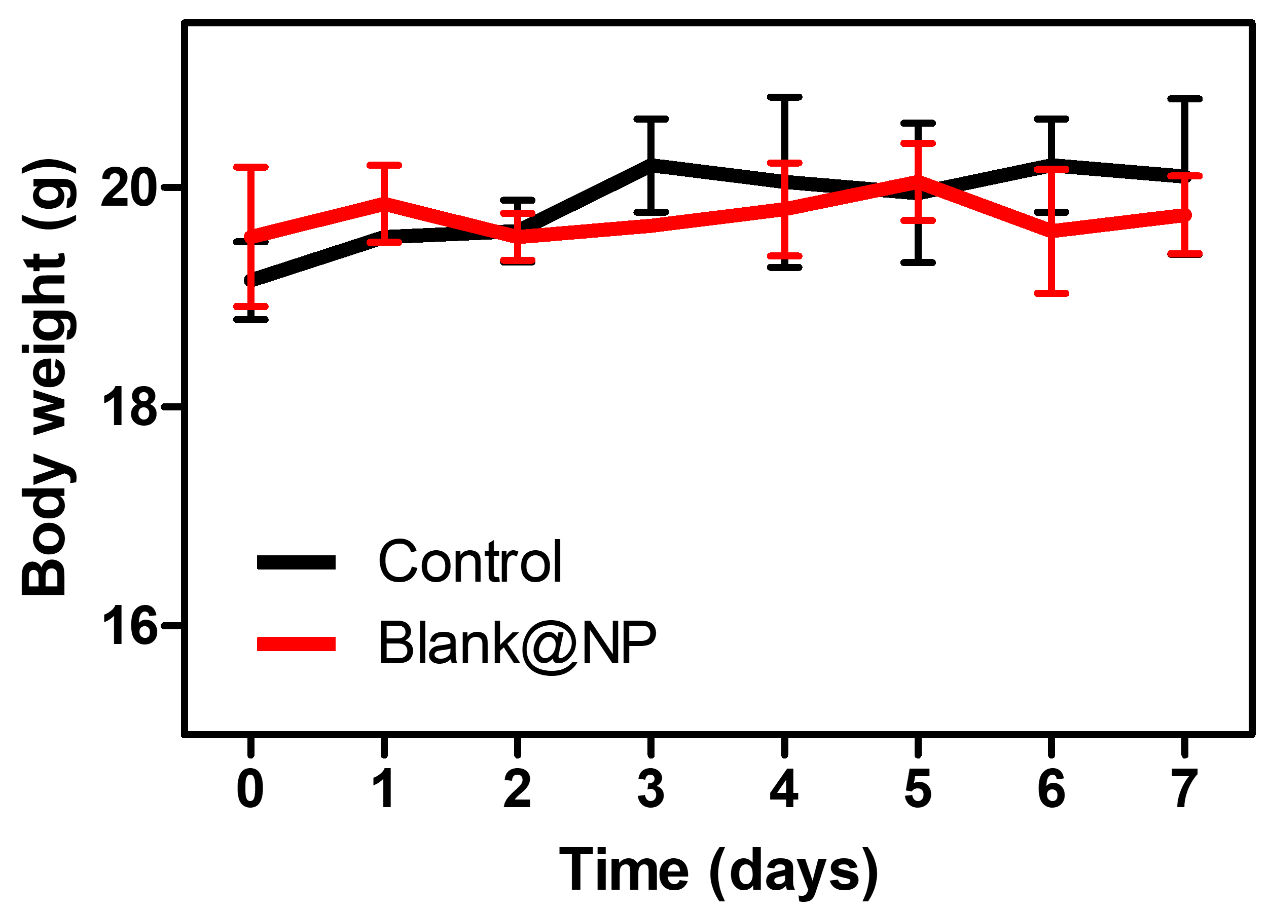
Figure S4. Changes of body weight over time in Control group and Blank@NP-treated group.**


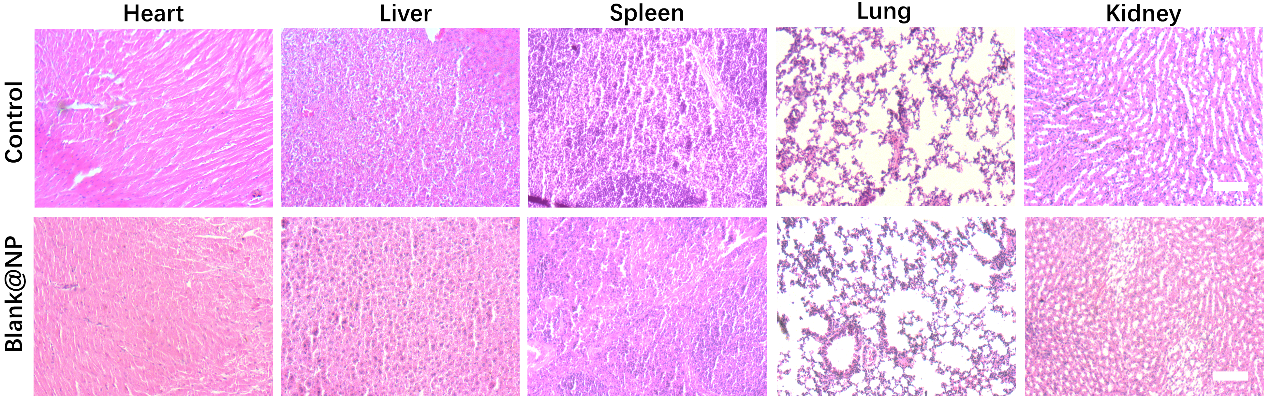


**Figure S5. *In vivo* toxicity was evaluated by H&E staining of vital organ tissues.** The organs were harvested, fixed in 10% formalin, embedded in paraffin, sectioned, subjected to H&E staining, and examined for histological assessment. Representative images are shown (n=3). Scale bar: 50 µm.


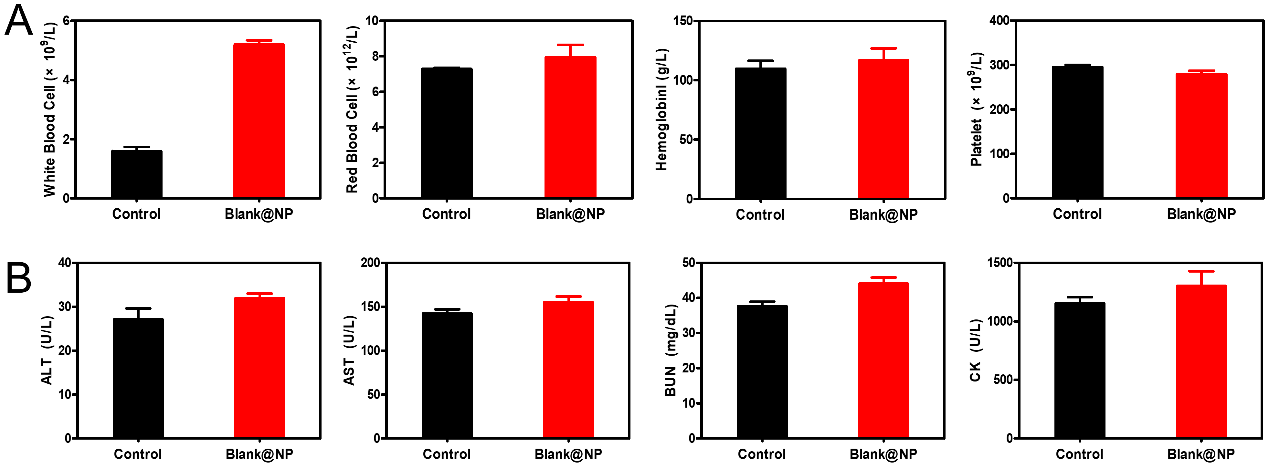


**Figure S6. Partial blood test (A) and biochemical parameters (B) of mice in the Control group and Blank@NP-treated group.** Each point represents the mean ± SEM (n = 3).


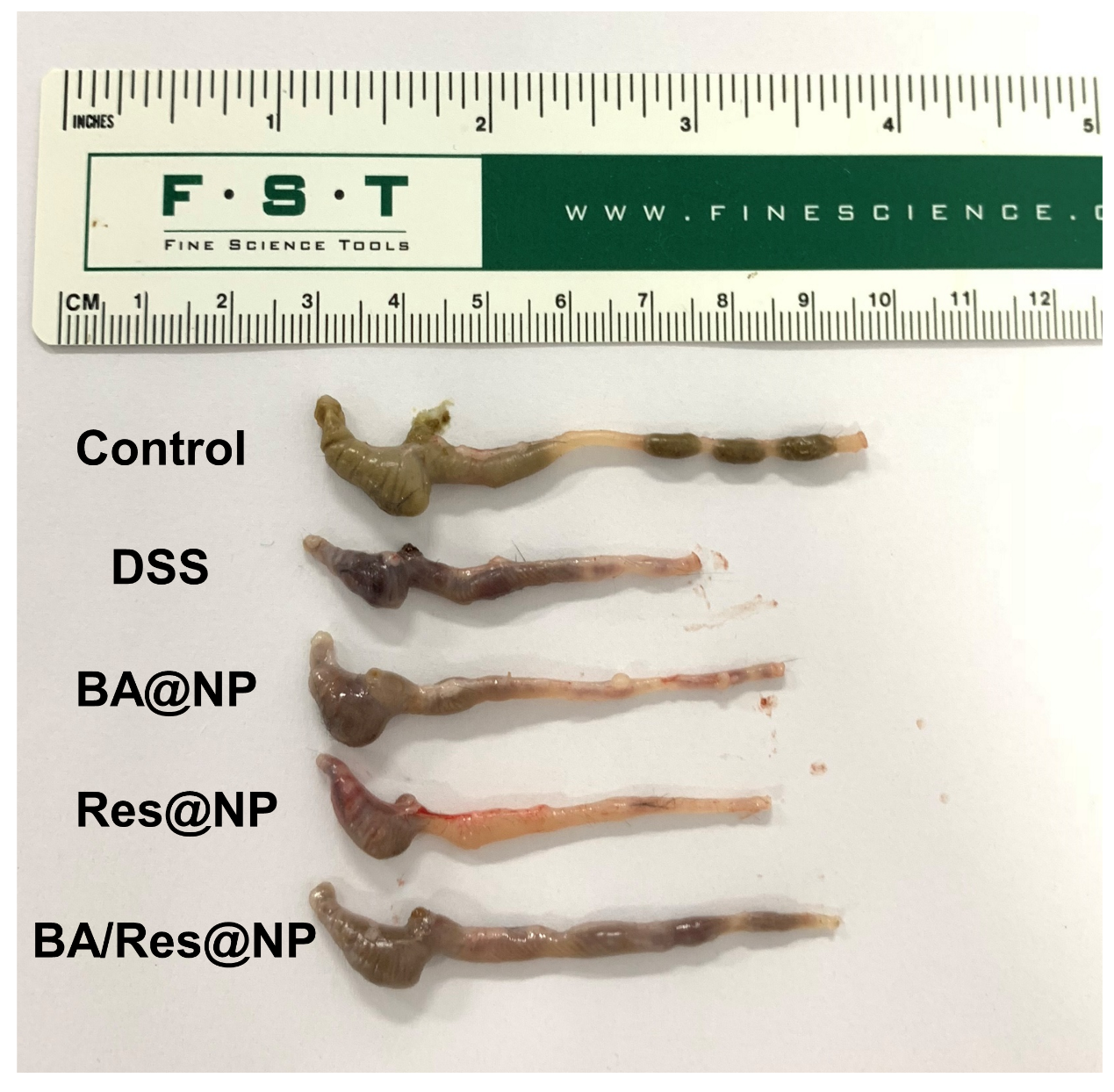


**Figure S7.** **Colon length in different groups of mice.**

**
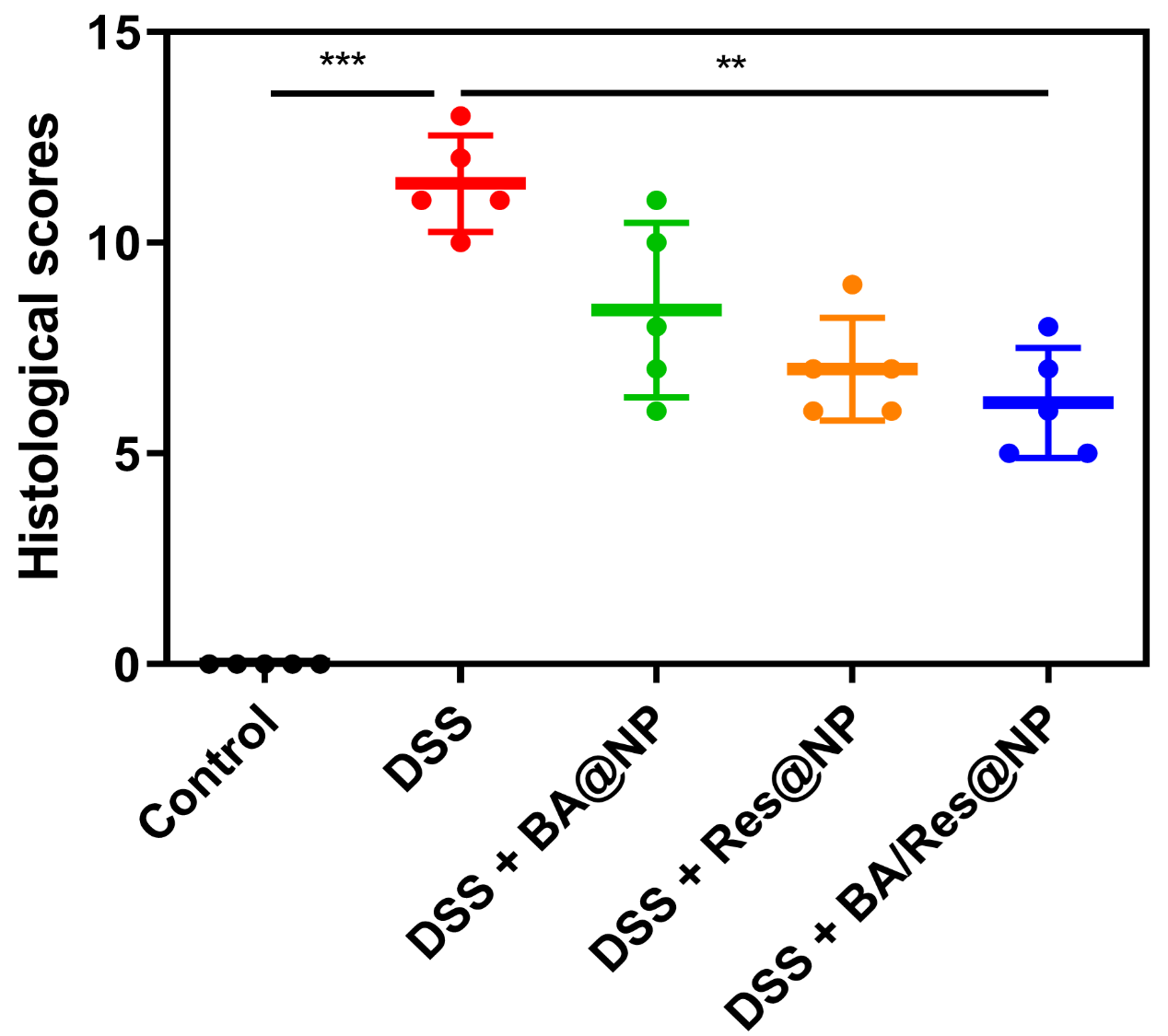
**

**Figure S8.** **Histopathologic score in different groups of mice.**

**Table S1. Primers used for Real-time PCR.**

| **Gene** | **Forward primer (5’-3’)** | **Reverse primer(5’-3’)** |
| --- | --- | --- |
| **TNF-*α*** | AGGCTGCCCCGACTACGT | GACTTTCTCCTGGTATGAGATAGCAAA |
| **IL-6** | ACAAGTCGGAGGCTTAATTACACAT | TTGCCATTGCACAACTCTTTTC |
| **IL-1*β*** | TCGCTCAGGGTCACAAGAAA | CATCAGAGGCAAGGAGGA AAA C |
| **IL-12** | GCCAGTACACCTGCCACAAA | TGTGGAGCAGCAGATGTGAGT |
| **36b4** | TCCAGGCTTTGGGCATCA | CTTTATCAGCTGCACATCACTCAGA |

**Table S2.** The entrapment efficiency and loading capacity of NPs doped with diﬀerent BA/Res ratios.

|  | Entrapment efficiency (%) | |  | Loading capacity (%) | | |
| --- | --- | --- | --- | --- | --- | --- |
| BA:RES(m:m) | BA | Res |  | BA | Res | |
| 1:0 | 56.841% | / |  | 32.720% | | / |
| 0:1 | / | 1.868% |  | / | | 5.348% |
| 1:1 | 69.355% | 1.792% |  | 46.241% | | 7.282% |
| 1:2 | 74.538% | 1.693% |  | 52.334% | | 8.708% |
| 2:1 | 66.613% | 1.093% |  | 44.412% | | 2.387% |

Notes: Data are presented as mean ± S.D. (n=3). Optimized batch was a ratio of 1:2.

**Additional methods**

**Staining of Colon Microarrays**

Normal colon tissue and tumor colon tissue microarrays (TMAs) (BC05002b) were obtained from US Biomax, Inc. (Derwood, MD, USA). The antigen was extracted and the slides were incubated with goat serum. The anti-P-selectin antibody was added and incubated for another 4 h, followed by a biotinylated secondary antibody for 1 h.

**Cell apoptosis study**

To quantify the cell viability and apoptosis of NP *in vitro*, we carried out Annexin V-FITC/propidium iodide (PI) apoptosis assay. Raw 264.7 macrophages were grown in 6-well plate with a 37 °C, 5% CO_2_ condition. After growing to a certain density, added the medium containing different NPs to each well. After the NPs were co-incubated with the cells for 6 h, LPS was used to induce inflammation in macrophages. Finally, cells were collected and washed twice with PBS buffer for flow cytometry. Before flow cytometry, added 5 μL annexin V-FITC to the cell suspension, and reacted at room temperature for 15 min avoiding light. Then 5 μL PI was added, mixed with cell suspension and reacted for 15 min. Finally, flow cytometry was performed on each group of cells, using different channels for Annexin V-FITC/ PI apoptosis assay.

***In vivo* monitoring of inflammation during acute UC.**

*In vivo* imaging system (IVIS) was used to detect the bioluminescence of mice. Before imaging, the C57BL/6 mice were depilated at the image site. The mice were intravenously injected with 100 μL of a mixture of luminol and DiL/DiD-loaded BA/Res@NP 1 h before imaging. After gas anesthesia, in vivo imaging was performed in supine position to obtain images. The bioluminescent images are taken every 2 min.

**Histological analyses of tissue sections by hematoxylin and eosin staining**

Fresh tissues were fixed in 10% formalin solution to prevent tissue deformation. Before the experiment, the fixed tissues were dehydrated and transparent, and the alcohol in the tissues was replaced with xylene for subsequent paraffin embedding. Soaked the tissues in melted paraffin wax and stored overnight. The tissues were paraffin embedded and then sectioned. Paraffin tissue blocks were sliced into thin slices with a thickness of 6 μm. The sliced slices are generally wrinkled and should be ironed in heated water, then pasted on slides and dried in a 45 ℃ incubator. Dewaxing of tissue sections was then performed. Before staining, paraffin sections were dewaxed with xylene and ethanol at different concentrations. Eosin stained the cytoplasm red and hematoxylin stained the nucleus blue. Tissue sections were observed and photographed under a microscope. Histological analysis and statistics of tissue sections were performed.

**Impact of BA/Res@NP on intestinal microbiota**

On the last day of experiment, collected the feces from mice in different groups to determine the microbiota using the 16S rRNA sequencing method in Beijing novogene Technology Co. Ltd (Beijing, China). 338F (5′-ACT CCT ACG GGA GGC AGC AG-3′) and 806R (5′-GGA CTA CHV GGG TWT CTA AT-3′) were used as the broadly conserved primers to amplify this region. PCR amplification, library preparation, library quality inspection and quantification were carried out for qualified environmental microbial DNA samples in the designated area, and the samples were differentiated with the set TAG sequence. Illumina Hiseq 2500 high-throughput sequencing platform was used to sequencing qualified libraries. Finally, data analysis was performed by Novomagic, which can carry out OTU species annotation, sample complexity analysis, multi-sample comparative analysis, environmental factor association analysis and so on. Each group contained at least 3 samples for 3 biological replicates.
